# Deep-NIR to NIR-II hemicyanine fluorophore scaffolds with dual optically tunable sites for *in vivo* multiplexed imaging

**DOI:** 10.1039/d5sc06690e

**Published:** 2025-11-03

**Authors:** Qinian Liu, Zhuoyang Li, Yujie Huang, Zhenni Lin, Xing-Can Shen, Hua Chen

**Affiliations:** a Key Laboratory for Chemistry and Molecular Engineering of Medicinal Resources (Ministry of Education of China), Guangxi Key Laboratory of Chemistry and Molecular Engineering of Medicinal Resources, School of Chemistry and Pharmaceutical Sciences, Guangxi Normal University Guilin 541004 P. R. China chenhuagnu@gxnu.edu.cn xcshen@mailbox.gxnu.edu.cn

## Abstract

Dual-locked probes are highly promising for use in medical diagnosis and drug screening due to their enhanced specificity and multiplex detection capabilities. However, most near-infrared (NIR) fluorophores used for dual-locked systems exhibit spectral activity in the blue portion (650–800 nm) of the NIR window, substantially restricting their application in deep-tissue imaging. Herein, for the first time, we report a novel class of deep-NIR to NIR-II hemicyanine fluorophore (Guilin, GL) scaffolds with dual optically tunable sites for dual-locked probe development. These GL dyes, obtained through chloride-substituted cyanine/hemicyanine hybridization, exhibit emission wavelengths spanning 800–950 nm and contain critical *meso*-Cl and hydroxyl/amino groups, which can be readily converted into two distinct reactive sites for multiplexed imaging analysis. To demonstrate the applicability of GL dyes, we engineered a novel dual-locked probe GL-Cys, which exhibited enhanced emission at 830 nm at low Cys levels and a distinct 765 nm signal elevation at elevated concentrations. More importantly, the GL-Cys probe enables effective differentiation of Cys levels in pancreatic/breast cancer models and corresponding tumor ferroptosis models through *in vivo* dual-channel ratiometric imaging. Thus, this molecular design paradigm establishes a versatile deep-NIR to NIR-II hemicyanine scaffold that may be generalized for multiplexed imaging of other analytes.

## Abstract 2

We have described a library of innovative deep-NIR to NIR-II hemicyanine fluorophore scaffolds (Guilin, GL) with dual optically tunable sites for dual-locked probe development.

## Graphical abstract

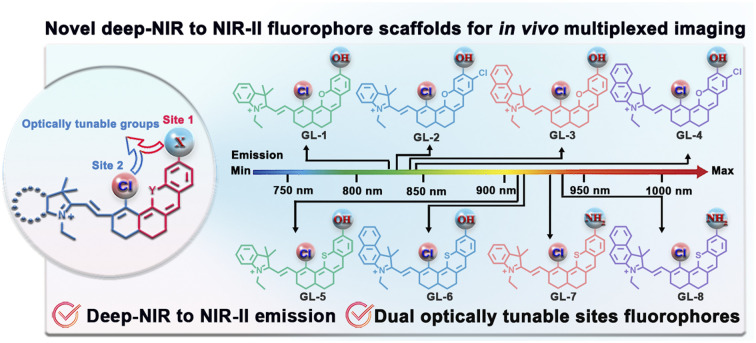

## Introduction

Unimolecular dual-locked probes have attracted significant attention in medical diagnosis, treatment, and drug screening due to their reduced signal crosstalk, enhanced specificity, increased spatial resolution and multiplex detection capabilities.^1–3^ Their ability to facilitate real-time simultaneous imaging of multiple biomarkers allows the exploration of the fundamental correlations between biomarkers and their differentiation within the pathological pathways of living organisms.^4–6^ This, in turn, offers a more profound comprehension of the underlying pathological mechanisms. Moreover, when combined with deep-near-infrared (deep-NIR) to NIR-II multiplexed fluorescence imaging with higher resolution and greater penetration depth, dual-locked probes have emerged as a powerful and promising tool for biological and biomedical research.^7–10^ Consequently, the design and development of smart dual-locked probes in the deep-NIR to NIR-II region is crucial for improving the accuracy of disease diagnosis and detection.

Currently, significant progress has been achieved in the development of dual-locked fluorescent probes, and design strategies for these probes typically fall in the following two categories.^1,11,12^ The first classic approach uses two dyes based on the Förster resonance energy transfer (FRET) mechanism, enabling detection of two distinct analytes through energy transfer between a donor and an acceptor.^13–15^ The second reported approach uses a single dye with dual optically tunable groups.^16,17^ In comparison, single-dye dual-locked probes possess several distinct advantages. For example, single-dye dual-locked probes typically have a simpler design, are more straightforward to synthesize and modify, and effectively eliminate the risk of inter-dye interference that may occur in multi-dye setups.^18,19^ To obtain such probes, some hybrid NIR dyes with dual optically tunable sites have been developed, significantly advancing single-dye dual-locked probes.^20–25^ However, most current fluorophores for these probes are restricted to the blue region of the near-infrared window (650–800 nm), limiting their deep tissue imaging applications.^26–29^ To address this issue, many researchers have focused on the development of deep-NIR to NIR-II fluorophores for *in vivo* imaging.^30–32^ However, due to the lack of multiple optically tunable sites, the design of single-dye dual-locked probes based on the reported dyes is quite challenging. To the best of our knowledge, almost no reports on deep-NIR to NIR-II fluorophores with dual optically tunable sites, and particularly with the controllable hydroxyl or amino groups, have been reported to date, owing to the challenge posed by the complexity of the molecular design. Therefore, despite its desirability, the development of deep-NIR to NIR-II fluorophores with dual optically tunable moieties for the design of dual-lock fluorescent probes remains challenging.

**Scheme 1:**
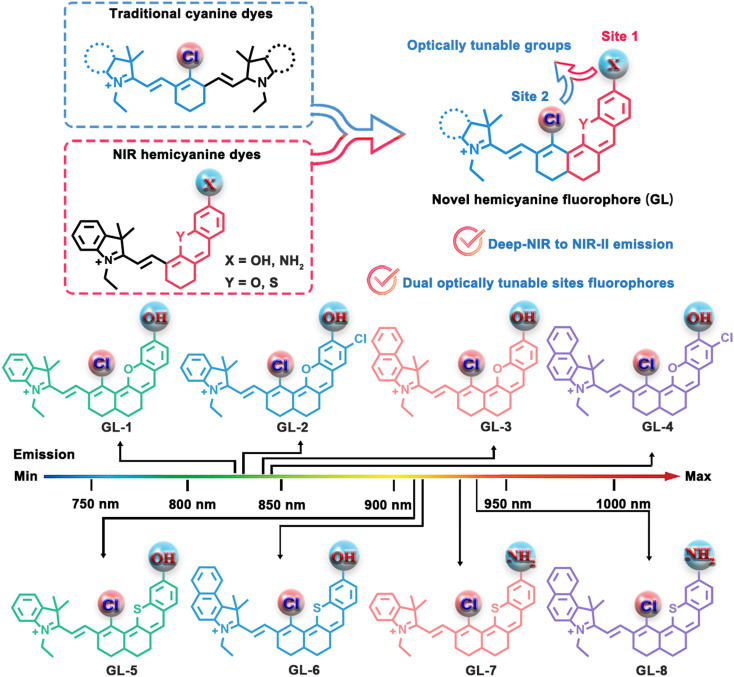
A library of innovative deep-NIR to NIR-II hemicyanine fluorophores with dual optically tunable sites for dual-locked probe development.

In this study, for the first time, we introduce a library of innovative deep-NIR to NIR-II hemicyanine fluorophores (named Guilin, GL) that integrate dual optically tunable sites, establishing a versatile platform for the development of dual-lock probes (Scheme 1). The designed GL dyes are achieved through classical chloride-substituted cyanine/hemicyanine hybridization, exhibit emission wavelengths ranging from 800 to 950 nm and bear crucial *meso*-Cl and hydroxyl/amino functional groups, enabling multiplexed imaging analysis. Based on these fluorophore scaffolds, we have engineered a novel dual-locked probe GL-Cys with Cys concentration-dependent spectral toggling. Specifically, this probe demonstrated enhanced emission at 830 nm at low Cys levels and a distinct increase in the 765 nm signal at higher Cys concentrations. Crucially, GL-Cys enabled effective differentiation of Cys levels in pancreatic/breast cancer mice models, as well as in relevant tumor ferroptosis models, highlighting the potential of GL fluorophores for multiplexed bioanalysis applications.

## Results and discussion

### Design, synthesis and photophysical properties of deep-NIR to NIR-II hemicyanine fluorophores, GL

**Fig. 1:**
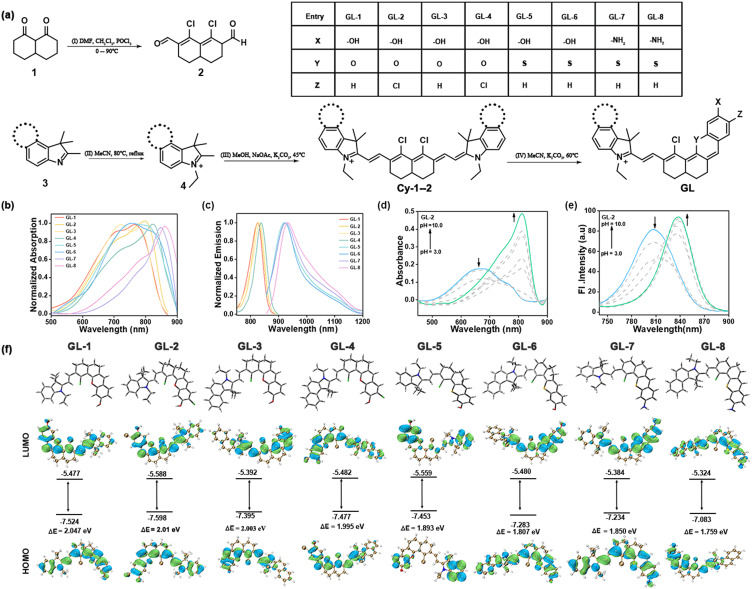
(a) Synthetic routes for GL. Reagents and conditions: (I) DMF, CH_2_Cl_2_, POCl_3_, 0–90 °C, 4 h. (II) MeCN, 80 °C, reflux, 12 h. (III) MeOH, NaOAc, K_2_CO_3_, 45 °C, 4 h. (IV) MeCN, K_2_CO_3_, 60 °C, 2 h. (b) Normalized absorption and (c) fluorescence spectra of GL-1–8 in MeOH. (d) and (e) pH-dependent absorbance and fluorescence emission spectra of GL-2 in MeCN/PBS (1 : 1 (v/v), pH = 7.4). (f) Theoretical calculations of the highest occupied molecular orbital (HOMO)/lowest unoccupied molecular orbital (LUMO) energy levels for GL-1–8 based on density functional theory (DFT) calculations at the B3LYP/6-31 + G(d) level.

Generally, hybridization is a key molecular engineering strategy that endows fluorophores with complementarity and multifunctionality. To obtain deep-NIR to NIR-II fluorophores featuring dual optically tunable sites, we sought to hybridize chloro-substituted cyanine with hemicyanine, producing an innovative type of NIR dyes (GL-1–8) with chlorine and hydroxyl/amino optically tunable groups. GL-1–8 dyes were synthesized *via* the classic hemicyanine synthesis method in good yield, and the synthetic routes for GL-1–8 used in this study are shown in Fig. 1a and SI Schemes S1–S10. All compounds were verified by nuclear magnetic resonance (NMR) spectroscopy (^1^H and ^13^C) and high-resolution mass spectroscopy (shown in the SI).

Next, the photophysical properties of GL-1–8 were determined in different solvents, including CH_2_Cl_2_, CH_3_OH, MeCN and MeCN/PBS (1 : 1 (v/v); pH = 7.4). The UV absorption and fluorescence spectra of GL-1–8 and the corresponding photophysical data are compiled in Fig. S1 and Tables S1–S4. As expected, the maximum absorption peaks of GL-1–8 dyes in MeOH were observed at 766, 799, 780, 824, 753, 764, 853, and 869 nm, respectively. Correspondingly, these dyes also exhibited strong fluorescence in the deep-NIR and NIR-II regions, with maximum emission peaks at 825, 827, 832, 839, 920, 923, 927, and 936 nm, respectively, suggesting their potential for deep-tissue imaging (Fig. 1b and c). To provide a theoretical basis for understanding the effects of different donors and acceptors on spectral shifts, the structures of GL-1–8 were optimized and analyzed using the Gaussian 9 software package, and the results were further processed using the Multiwfn and VMD programs.^33,34^ Density functional theory (DFT) calculations show that GL-1–8 displayed gradually decreasing energy gaps from 2.047 eV to 1.759 eV, in good agreement with the experimental spectral data (Fig. 1f). Given the complex and variable biological milieu where oxidizing agents and nucleophiles can affect dye stability, we tested GL-1–8 against common biological interferents, namely glutathione (GSH), NaClO, Na_2_S, and H_2_O_2_ in MeCN/PBS (1 : 1 (v/v); pH = 7.4). As shown in Fig. S2 and S3, the absorption and fluorescence spectra of GL-1–8 remained essentially unchanged under all conditions, strongly indicating the good chemical stability of GL-1–8 under physiological conditions.

To support the design concept of GL-1–8 dyes with dual optically tunable sites, we validate the optical tuning capabilities of the *meso*-Cl and hydroxyl/amino functional groups, respectively. First, these dyes were subjected to a range of pH levels to mimic the protonation and deprotonation of phenolic compounds. As expected, the emission spectra of GL-1–6 (Fig. 1d, e and S4) under different pH conditions exhibited an increased fluorescence band centered at *λ* = 820–940 nm as well as a decreased fluorescence band centered at *λ* = 740–860 nm. Moreover, the absorption spectra also displayed pH-induced wavelength shifts. To demonstrate that the amino group is an optically tunable site, we synthesized GL-Ac as control through the acetylation of GL-7 with acetic anhydride (Scheme S11). As expected, GL-Ac exhibited a significant blue-shift compared to GL-7, with maximum absorption in MeOH at 849 nm and 700 nm for GL-7 and GL-Ac, respectively (Fig. S5). The above results indicate that the hydroxyl/amino functional groups can effectively regulate the optical properties of the GL-1–8 dyes. Furthermore, to validate the feasibility of the *meso*-Cl as a tunable site, we designed a thiol probe GL-OH by modifying the *meso*-Cl with 3,5-bis(trifluoromethyl)benzenethiol (Scheme S12). Upon reaction with Cys, the probe displayed a significant blue shift in the absorption spectra (Fig. S6). To further validate the feasibility of dual optically tunable sites in GL dyes, we performed Gaussian calculations for GL-7/GL-Ac and GL-OH/GL-OH-Cys. The results demonstrate that upon acetylation of GL-7 to form GL-Ac, the energy gap increased slightly from 1.850 eV to 1.856 eV. The enlargement of the LUMO–HOMO gap correlates with the blue shift observed for GL-Ac, confirming that the amino group can serve as an effective optically tunable site. Moreover, GL-OH-Cys exhibited a significant change in the energy gap compared to GL-OH, increasing from 1.934 eV to 2.119 eV. The expansion of the LUMO–HOMO gap correlates well with the blue shift observed in GL-OH-Cys, demonstrating that the *meso*-Cl can function as an effective molecular optically tunable site (Fig. S7). All of these results demonstrated that GL dyes can be used as excellent deep-NIR to NIR-II fluorophores with dual optically tunable sites.

### Design, synthesis and photophysical properties of the dual-locked probe GL-Cys

**Scheme 2:**
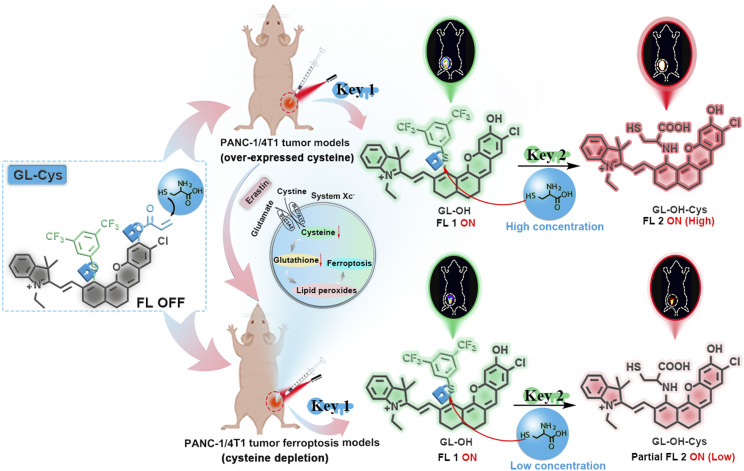
Schematic illustration of the dual-lock fluorescent probe for dual-channel differentiation of Cys levels in tumor mice models and corresponding tumor ferroptosis models.

Given the outstanding performance of dual optically tunable sites in deep-NIR to NIR-II hemicyanine fluorophore (GL) scaffolds, using GL-2 as a scaffold, we for the first time constructed the dual-locked probe GL-Cys by integrating distinct Cys-reactive sites (3,5-bis(trifluoromethyl)benzenethiol and acryloyl groups)^35,36^ for effective differentiation of Cys levels in pancreatic/breast tumor mice models and corresponding tumor ferroptosis models *in vivo* (Scheme 2). Detailed synthesis procedures and characteristic data for GL-Cys are provided in the SI (Scheme S13).

**Fig. 2:**
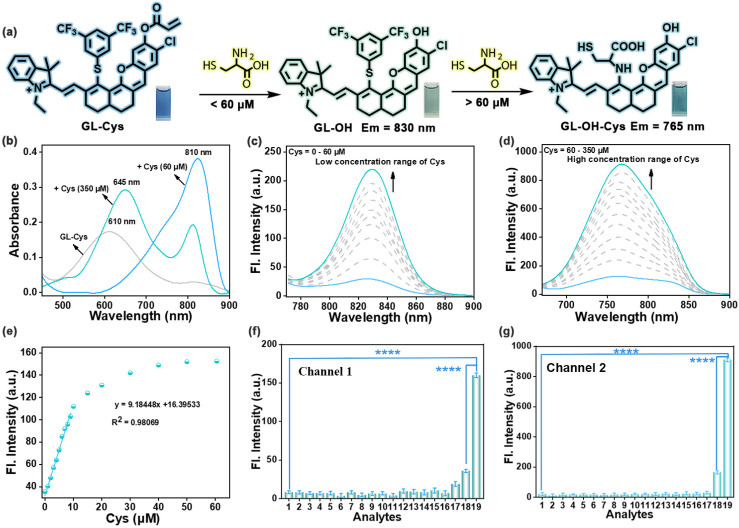
(a) Mechanism of GL-Cys for detection of different Cys concentrations. (b) UV absorption spectra of GL-Cys (10 μM) responding to different Cys concentrations. (c) and (d) Fluorescence emission spectra of GL-Cys (10 μM) with different amounts of Cys: (c) 0–60 μM, *λ*_ex_ = 720 nm, and *λ*_em_ = 830 nm; (d) 60–350 μM, *λ*_ex_ = 640 nm, and *λ*_em_ = 765 nm). (e) Plot of emission intensity at 830 nm *versus* Cys content. (f and g) Channel 1 (*λ*_em_ = 830 nm) and channel 2 (*λ*_em_ = 765 nm) fluorescence intensity changes of GL-Cys (10 μM) after incubation for 25 min with different species: (1) blank (10 μM GL-Cys); (2) 100 μM alanine (Ala); (3) 100 μM Na_2_S; (4) 100 μM methionine (Met); (5) 100 μM proline (Pro); (6) 200 μM H_2_O_2_; (7) 100 μM tyrosine (Tyr); (8) 100 μM Na_2_S_2_O_3_; (9) 100 μM NaCl; (10) 100 μM NaClO; (11) 100 μM NaNO_2_; (12) 100 μM tryptophan (Trp); (13) 100 μM threonine (Thr); (14) 100 μM valine (Val); (15) 100 μM ZnCl_2_; (16) 100 μM CaCl_2_; (17) 1 mM GSH; (18) 100 μM Hcy; (19) 60 μM Cys (channel 1)/350 μM Cys (channel 2). Error bars represent standard deviation (SD) of three experiments. Statistical analysis was performed using one-way ANOVA and multiple comparison tests of significant differences (****p <0.0001).

To investigate the feasibility of GL-Cys to distinguishably detect Cys, the changes in the absorption spectrum upon reaction of GL-Cys with Cys were evaluated (Fig. 2). When low concentrations of Cys (0–60 μM) were added, the absorbance of the GL-Cys solution at 610 nm gradually decreased. Subsequently, a new absorption band and a new emission band emerged at 810 nm and 830 nm, respectively, and the color of the solution gradually shifted from blue to green (Fig. 2a and c). Notably, addition of excess Cys induced a pronounced blue shift in the absorption spectrum from 810 nm to 645 nm, accompanied by a corresponding color change from green to turquoise. Across the Cys concentration range of 60–350 μM, a progressive enhancement in the fluorescence intensity was observed at 765 nm (Fig. 2d). Furthermore, the time-dependent response of GL-Cys to Cys indicated that after adding low concentrations of Cys (60 μM), equilibrium was reached within 100 s (Fig. S8a). The high-resolution mass spectrometry (HRMS) results revealed that after mixing the GL-Cys solution with Cys, characteristic signals at *m*/*z* 782.1950 and *m*/*z* 728.1844 were detected and identified as GL-Cys and GL-OH, respectively (Fig. S8b). These results indicated that Cys attacks the acrylate group of GL-Cys through a Michael addition reaction to form a sulfide, and intramolecular cyclization releases GL-OH. Notably, a significant linear correlation was observed between the fluorescence intensity of GL-Cys and Cys concentration (0–10 μM). Based on the fluorescence spectrum, the detection limit (3*σ*/*k*) for Cys using GL-Cys was approximately 31.3 nM (Fig. 2e). In addition, dynamic changes in the fluorescence spectrum indicated that the GL-Cys probe completely reacted with Cys within 25 min at high concentration (350 μM) (Fig. S8c). According to previous reports,^37,38^ this prolonged reaction time was attributed to the GL-Cys probe first undergoing a Michael addition reaction to release GL-OH. Moreover, the phenylene sulfur group in GL-OH, which are typical nucleophilic substitution leaving groups, were covalently attached to the fluorophore to generate GL-OH-Cys. Concurrently, HRMS revealed characteristic signals at *m*/*z* 782.1954, *m*/*z* 728.1847, and *m*/*z* 603.2102 for GL-Cys, GL-OH, and GL-OH-Cys, respectively (Fig. S8d). DFT calculations were performed for both GL-Cys and GL-OH. As displayed in Fig. S9, the LUMO–HOMO energy gaps (Δ*E*) of GL-Cys and GL-OH were 1.981 and 1.934 eV, respectively.

These gaps showed a decreasing trend, which explained the changes in the absorption wavelength. These results indicate that the dual-locked probe GL-Cys is effective for both low and high concentrations of Cys with different emission channels.

To validate the applicability of the probe in complex biological matrices, selectivity experiments were conducted to assess the response of GL-Cys to various reactive species. In channel 1 (*λ*_em_ = 830 nm), only the Cys group induced a 7.8-fold increase in the fluorescence intensity, while other substances such as reactive sulfur/oxygen/nitrogen species, amino acids, cations, and anions did not cause a significant fluorescence enhancement (Fig. 2f). Similarly, in channel 2 (*λ*_em_ = 765 nm), only Cys addition led to a 12.7-fold fluorescence intensity increase (Fig. 2g). These results indicate that GL-Cys shows higher selectivity for Cys than other tested biological species, making it suitable for specific dual-channel Cys recognition and high-fidelity imaging in biological systems.

### Fluorescence imaging of the dual-locked probe GL-Cys in various cell lines *in vitro*

Prior to utilizing GL-Cys for fluorescence imaging in living cells, the biocompatibility of GL-Cys was investigated across various cell lines, such as PANC-1, 4T1, and L929 cells. Notably, the corresponding cell viability exceeded 80% when the concentration of GL-Cys reached 25 μM (Fig. S10), indicating its low cytotoxicity and potential for future *in vivo* applications. Subsequently, the subcellular organelle-targeting ability of GL-Cys was verified using various commercial organelle trackers. The fluorescence images of the probe showed good overlap with those of the commercial mitochondrial reagent within PANC-1 cells and 4T1 cells, indicating that GL-Cys has good mitochondrial targeting ability (Fig. S11).

**Fig. 3:**
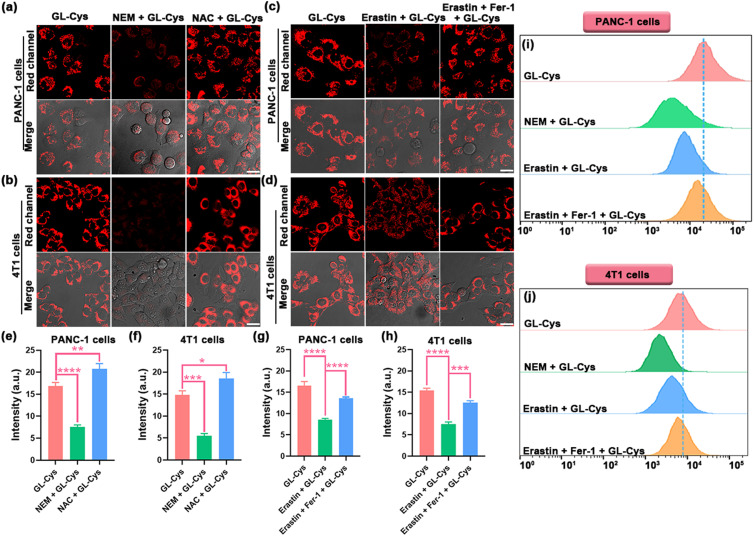
Confocal imaging of GL-Cys in PANC-1 cells and 4T1 cells under various pretreatment conditions. The PANC-1 cells (a) and 4T1 cells (b) were divided into three groups: (1) GL-Cys only; (2) pretreated with NEM (100 μM) for 30 min, followed by incubation with the probe for another 30 min; (3) pretreated with NAC (100 μM) for 30 min, followed by incubation with the probe for another 30 min. The PANC-1 cells (c) and 4T1 cells (d) were categorized into three experimental groups: (1) GL-Cys only; (2) incubated with erastin for 8 h, followed by incubation with GL-Cys for another 30 min; (3) incubated with erastin for 8 h, followed by treatment with ferrostatin-1 (Fer-1) for 2 h and by subsequent addition of the GL-Cys for another 30 min. (e–h) Quantitative data for the relative fluorescence intensity of images in (a–d). (i) and (j) Results of flow cytometry experiments used to assess ferroptosis in PANC-1 cells and 4T1 cells. Scale bars: 20 μm. Error bars represent standard deviation (SD) of three experiments. Statistical analysis was performed using one-way ANOVA and multiple comparison tests of significant differences (*p <0.05, **p <0.01, ***p <0.001, and ****p <0.0001).

Subsequently, PANC-1 cells and 4T1 cells were incubated with GL-Cys to confirm the ability of the probe to effectively distinguish varying Cys levels. As depicted in Fig. 3a, b, e and f, endogenous Cys triggered red fluorescence emission in the cells. When PANC-1 and 4T1 cells were pre-treated with *N*-ethylmaleimide (NEM), a biothiol scavenger, for 30 min before incubation with GL-Cys, a marked reduction in red fluorescence was observed, indicating diminished intracellular Cys levels. By contrast, pre-treatment of the cells with *N*-acetyl-l-cysteine (NAC), a cysteine donor, for 30 min prior to incubation with GL-Cys resulted in a significant enhancement of red fluorescence, implying that NAC supplementation effectively augmented intracellular Cys levels. Collectively, these findings demonstrate that the probe GL-Cys can accurately detect both elevated and reduced concentrations of Cys within cells.

Cysteine (Cys) is a reliable biomarker for assessing the severity of malignant tumors, particularly pancreatic cancer, and the occurrence of tumor ferroptosis is also closely related to the Cys levels.^39,40^ Erastin is a cell-permeable ferroptosis activator that has been widely regarded to be an effective agent for inducing ferroptosis.^41–43^ Consequently, further investigation was conducted to explore the suitability of the probe GL-Cys for tracking Cys level fluctuations during ferroptosis. PANC-1 cells and 4T1 cells were pre-incubated with erastin for 8 h and then treated with GL-Cys for 30 min, leading to reduced red-channel fluorescence intensity. Fer-1 can alleviate erastin-induced cytoplasmic and lipid accumulation of reactive oxygen species.^44–47^ Subsequently, PANC-1 cells and 4T1 cells were incubated with erastin for 8 h and then treated with Fer-1 for 2 h, followed by 30 min incubation with GL-Cys. Compared to the cells treated with erastin only, red-channel fluorescence signals of the cells treated with erastin and Fer-1 slightly increased (Fig. 3c, d, g and h), indicating elevated Cys levels in the ferroptosis cell model. Furthermore, these results were corroborated through flow cytometry analysis (Fig. 3i and j). These findings suggest that GL-Cys can effectively monitor dynamic changes in the Cys levels during ferroptosis in tumor cells.

### Visualizing Cys levels during ferroptosis in pancreatic cancer through *in vivo* dual-channel fluorescence imaging

Prior to *in vivo* fluorescence imaging, we first evaluated the fluorescence imaging capability of GL-Cys in MeCN/PBS (1 : 1 (v/v); pH = 7.4) solutions in the presence of low/high concentration range of Cys using the Kodak *in vivo* FX Pro imaging system (Bruker), revealing the emergence of significant differences in the fluorescence intensities of the two channels. Initially, no fluorescence signals were detected prior to the addition of Cys. However, following the introduction of low-concentration Cys (Fig. S12a), fluorescence emission was predominantly observed in channel 1 (*λ*_ex_ = 720 nm with a filter at *λ* = 790 nm), with a 2.91-fold fluorescence intensity increase. Interestingly, upon introduction of excess Cys (Fig. S12b and c), fluorescence emission mainly shifted to channel 2 (*λ*_ex_ = 690 nm, with a filter at *λ* = 750 nm), causing an 8.50-fold fluorescence intensity increase. These results demonstrate that changes in the Cys concentration induce significant channel-specific fluorescence changes, confirming the efficacy of GL-Cys for dual-channel *in vitro* Cys detection.

**Fig. 4:**
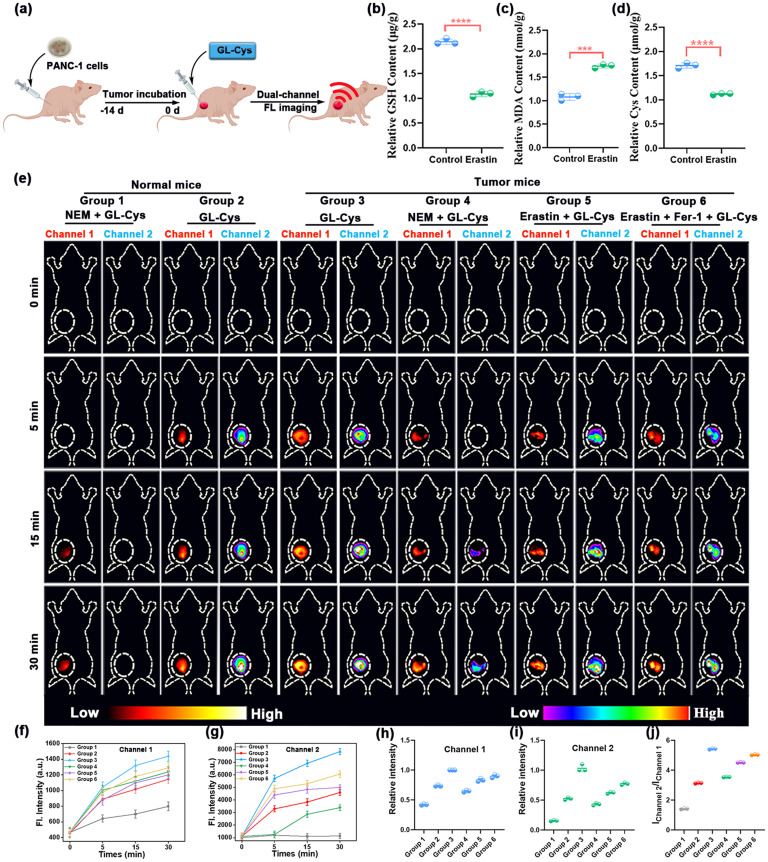
(a) Schematic illustration of GL-Cys in Cys-based dual-channel imaging in a subcutaneous PANC-1 tumor mice model. Determination of (b) GSH, (c) MDA and (d) Cys content in the PANC-1 tumor of mice induced with erastin to undergo ferroptosis. (e) Time-dependent dual-channel fluorescence imaging of different groups of mice under different treatments (channel 1: *λ*_ex_ = 720 nm with a filter at *λ* = 790 nm; channel 2: *λ*_ex_ = 690 nm, with a filter at *λ* = 750 nm). Group 1: normal mice, NEM + GL-Cys; Group 2: normal mice + GL-Cys; Group 3: PANC-1 tumor mice, GL-Cys; Group 4: PNAC-1 tumor mice, NEM + GL-Cys; Group 5: PNAC-1 tumor mice, erastin + GL-Cys; Group 6: PNAC-1 tumor mice + erastin + Fer-1 + GL-Cys. (f–i) Corresponding fluorescence intensities of Groups 1–6. (j) Fluorescence ratio values (channel 2/channel 1) at 30 min for different groups taken from (e). Error bars represent standard deviation (SD) of three experiments. ***p <0.001; ****p <0.0001; Student's *t*-test.

The successful *in vitro* simulation of Cys activation provides a firm basis for real-time monitoring Cys levels in living organisms. To realize *in vivo* Cys level detection, we extensively evaluated the toxicity of the probe GL-Cys. Hematoxylin and eosin (H&E) staining revealed no observable pathological alterations in major organs (heart, liver, lungs, spleen, and kidneys) of GL-Cys-treated mice compared to control, thereby demonstrating the excellent biosafety of the GL-Cys probe (Fig. S13). Subsequently, the *in vivo* metabolic kinetics of the probe GL-Cys were further evaluated. The experimental results demonstrated that GL-Cys predominantly accumulated in the liver within 70 min post-injection and was effectively cleared *via* the hepatobiliary pathway (Fig. S14a and b). *Ex vivo* organ imaging revealed no significant fluorescence distribution in the major organs, except for a weak residual signal in the liver (Fig. S14c and d). These metabolic characteristics indicate that GL-Cys can be effectively cleared through physiological pathways, providing important evidence for its favorable biocompatibility. Then, *in vivo* dual-channel fluorescence imaging of GL-Cys was systematically evaluated both in normal and pancreatic cancer mice. As demonstrated in Fig. 4, GL-Cys was administered *via* intradermal injection to both groups of normal mice (Group 1: pretreatment with NEM; Group 2: only probe). Fig. 4e reveals that compared to Group 2, the probe-injected area in Group 1 exhibited a significantly reduced signal, with a weak fluorescence signal in channel 1 and no detectable fluorescence signal in channel 2, indicating that the dual-locked probe GL-Cys can effectively distinguish different Cys levels using the fluorescence signals of the two channels.

To assess the imaging efficacy of GL-Cys in tumor mice, we established a subcutaneous pancreatic tumor model and observed the dual-channel fluorescence variations after subcutaneous injection of the probes over an identical time frame (Fig. 4a). The pancreatic tumor mice were divided into four groups. As shown in Fig. 4e, slight fluorescence enhancement in Group 3 was observed in the fluorescence signal of channel 1 compared to Group 2, whereas in channel 2 it increased approximately from 0.52 ± 0.02 to 1.0 ± 0.02, indicating that the Cys level in pancreatic cancer mice is significantly higher than that in normal mice. Upon treatment of pancreatic tumor mice with NEM (Group 4), we observed a decrease in the dual-channel signal to the level observed in Group 2 (Fig. 4h and i). The capability of the GL-Cys in visualizing differentiation of Cys levels in the pancreatic tumor model and tumor ferroptosis models *in vivo* was further demonstrated. Following a 48 h pretreatment of the tumor with erastin (Group 5), the subcutaneous injection of GL-Cys resulted in a slight decrease in the fluorescence signal in channel 1 compared to Group 3, whereas a significant decrease in the fluorescence intensity in channel 2 compared to Group 3, from approximately 1 ± 0.02 to 0.65 ± 0.03, was obtained (Fig. 4i), showing that pancreatic cancer ferroptosis in mice is accompanied by significant downregulation of Cys. Additionally, when the tumor underwent 48 h pretreatment with erastin, followed by 8 h treatment with Fer-1 prior to GL-Cys injection (Group 6), the fluorescence intensity in channels 1 and 2 increased slightly compared to Group 5. Given the significant alterations in tumor ferroptosis markers, we measured the levels of MDA, GSH, and Cys in tumor tissues. As illustrated in Fig. 4c, MDA, which is a marker of lipid peroxidation in tumors, exhibited a significant increase by approximately 1.8-fold.

By contrast, the GSH and Cys levels decreased (Fig. 4b and d), thereby confirming the successful establishment of a pancreatic cancer tumor ferroptosis model. As shown in Fig. 4j, the fluorescence ratio of channel 2 to channel 1 also reflects the level of Cys. The pancreatic cancer group showed the highest fluorescence ratio of channel 2 to channel 1 of approximately 5.45, while the ratio in the ferroptosis group (4.54) was significantly lower. The experimental results demonstrate that GL-Cys enables effective differentiation of Cys levels in pancreatic tumor models and tumor ferroptosis models through *in vivo* dual-channel ratiometric fluorescence imaging.

### *In vivo* dual-channel imaging of the probe GL-Cys in breast cancer mice models

**Fig. 5:**
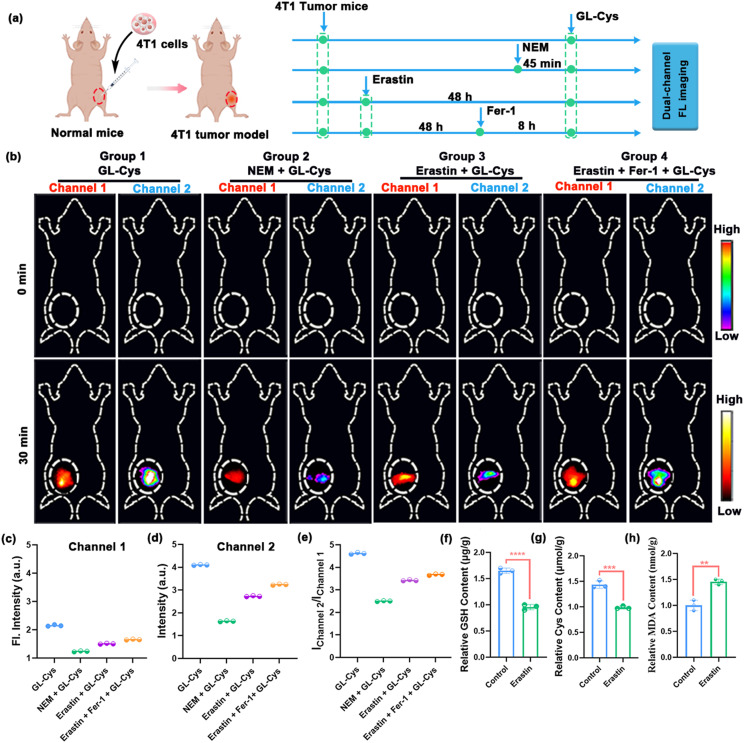
(a) Schematic illustration of the use of GL-Cys in dual-channel fluorescence imaging of Cys in 4T1 tumor mice. (b) Dual-channel fluorescence imaging of the 4T1 tumor model in different treatment groups at 0 and 30 min (channel 1: *λ*_ex_ = 720 nm with a filter at *λ* = 790 nm; channel 2: *λ*_ex_ = 690 nm, with a filter at *λ* = 750 nm). Group 1: 4T1 tumor mice, GL-Cys; Group 2: 4T1 tumor mice, NEM + GL-Cys; Group 3: 4T1 tumor mice, erastin + GL-Cys; and Group 4: 4T1 tumor mice + erastin + Fer-1 + GL-Cys. (c and d) Corresponding fluorescence intensities of different groups taken from (b). (e) NIR ratiometric analysis (channel 2/channel 1) at 30 min for different groups taken from (b). Measurement of GSH (f), Cys (g) and MDA (h) levels in the 4T1 tumor of mice induced with erastin to undergo ferroptosis. Error bars represent standard deviation (SD) of three experiments. **p <0.01; ***p <0.001; ****p <0.0001; Student's *t*-test.

Inspired by the excellent performance of GL-Cys in pancreatic tumor models, we further employed this probe for dual-channel fluorescence imaging of Cys in breast cancer tumor models. Subcutaneous breast tumor models were established using BALB/c nude mice, and the mice were then divided into four groups (Group 1: tumor mice + GL-Cys; Group 2: tumor mice + NEM + GL-Cys; Group 3: tumor mice + erastin + GL-Cys; Group 4: tumor mice + erastin + Fer-1 + GL-Cys) (Fig. 5a). Compared to Group 1, the dual-channel fluorescence intensity of Group 2 gradually decreased from 0 to 30 min (Fig. 5b), demonstrating that NEM induction downregulated Cys in 4T1 tumors. In the 4T1 ferroptosis induction Group 3, similar to the pancreatic cancer ferroptosis mice, we observed a significant decrease in the fluorescence intensity across both channel 1 (2.12 ± 0.03 to 1.25 ± 0.02) and channel 2 (4.12 ± 0.03 to 2.75 ± 0.02) compared to the tumor-bearing mice of Group 1, indicating that Cys is also downregulated during ferroptosis in 4T1 mice (Fig. 5c and d). After injecting the ferroptosis inhibitor Fer-1 in Group 4, the fluorescence signals in both channel 1 and channel 2 significantly increased compared to Group 3 (Fig. 5c and d). NIR ratiometric fluorescence analysis (channel 2/channel 1) also confirmed these results (Fig. 5e). To verify the occurrence of ferroptosis, an in-depth analysis of the tumors after different treatments was conducted, and the levels of Cys, GSH and MDA in the tumors were measured following various treatments using a commercial kit. The results demonstrated a decrease in both Cys and GSH levels, along with an increase in MDA levels (Fig. 5f–h), thereby confirming the erastin-induced 4T1 tumor ferroptosis model. The experimental results demonstrate that GL-Cys can effectively distinguish Cys levels between breast cancer tumor models and corresponding tumor ferroptosis models through *in vivo* dual-channel fluorescence imaging, underscoring the potential of GL fluorophores for multiplexed bioanalysis.

## Conclusion

In summary, this study successfully presents a new family of deep-NIR to NIR-II hemicyanine fluorophores (GL) featuring dual optically tunable sites for the development of dual-locked probes. These GL dyes, obtained through chloride-substituted cyanine/hemicyanine hybridization, exhibit emission wavelengths spanning 800–950 nm. Remarkably, these dyes integrate the optically tunable sites of cyanine and hemicyanine, and their optical properties can be easily tuned *via* the *meso*-Cl (like in cyanine dyes) and terminal amino/hydroxyl groups (like in hemicyanine dyes), thereby significantly advancing the development of dual-locked probes. Based on these fluorophores, we developed a smart dual-locked probe GL-Cys that demonstrated excellent responsiveness and selectivity to Cys *in vitro* and Cys concentration-dependent spectral switching, showing enhanced 830 nm emission at low Cys levels and increased 765 nm signals at high concentrations. More importantly, GL-Cys enables effective differentiation of Cys levels in various tumor models and their corresponding tumor ferroptosis models through *in vivo* dual-channel ratiometric fluorescence imaging, underscoring the potential of GL fluorophores as a versatile platform for multiplexed bioanalysis. Thus, the GL scaffold provides a firm basis for developing advanced probes targeting diverse analytes for NIR multicolor imaging in deep tissues, with future studies likely to expand its applicability to a broader spectrum of targets.

## Author contributions

The manuscript was written through contributions of all authors.

## Conflicts of interest

The authors declare no competing financial interest.

## Supplementary Material

SC-016-D5SC06690E-s001

## Data Availability

All data are available in the main text and the Supplementary Information (SI). Supplementary information: experimental procedures, characterization data, and supporting figures and tables. See DOI: https://doi.org/10.1039/d5sc06690e.

## References

[cit1] Wu L., Huang J., Pu K., James T. D. (2021). Nat. Rev. Chem..

[cit2] Dou K., Lu J., Xing Y., Wang R., Won M., Kim J., Yu F., Seung Kim J. (2025). Angew. Chem., Int. Ed..

[cit3] Schauenburg D., Gao B., Rochet L. N. C., Schüler D., Coelho J. A. S., Ng D. Y. W., Chudasama V., Kuan S. L., Weil T. (2024). Angew. Chem., Int. Ed..

[cit4] Kolanowski J. L., Liu F., New E. J. (2018). Chem. Soc. Rev..

[cit5] Zhu C., Han J., Liang F., Zhu M., Zhang G., James T. D., Wang Z. (2024). Coord. Chem. Rev..

[cit6] Wu L., Liu J., Tian X., Groleau R. R., Feng B., Yang Y., Sedgwick A. C., Han H.-H., Wang Y., Wang H.-M., Huang F., Bull S. D., Zhang H., Huang C., Zang Y., Li J., He X.-P., Li P., Tang B., James T. D., Sessler J. L. (2022). J. Am. Chem. Soc..

[cit7] Li J., Dong Y., Wei R., Jiang G., Yao C., Lv M., Wu Y., Gardner S. H., Zhang F., Lucero M. Y., Huang J., Chen H., Ge G., Chan J., Chen J., Sun H., Luo X., Qian X., Yang Y. (2022). J. Am. Chem. Soc..

[cit8] Chen Z., Li Q., Wu Y., Liu J., Liu L., Su L., Wu R., Song J. (2025). Nat. Commun..

[cit9] Chen L., Peng M., Ouyang Y., Chen J., Li H., Wu M., Qu R., Zhou W., Zhang C., Jiang Y., Xu S., Wu W., Jiang X., Zhen X. (2025). J. Am. Chem. Soc..

[cit10] Antaris A. L., Chen H., Cheng K., Sun Y., Hong G., Qu C., Diao S., Deng Z., Hu X., Zhang B., Zhang X., Yaghi O. K., Alamparambil Z. R., Hong X., Cheng Z., Dai H. (2016). Nat. Mater..

[cit11] Wu L., Li Z., Wang K., Groleau R. R., Rong X., Liu X., Liu C., Lewis S. E., Zhu B., James T. D. (2025). J. Am. Chem. Soc..

[cit12] Cheng P., Pu K. (2024). Chem. Soc. Rev..

[cit13] Wu L., Huang C., Emery B. P., Sedgwick A. C., Bull S. D., He X.-P., Tian H., Yoon J., Sessler J. L., James T. D. (2020). Chem. Soc. Rev..

[cit14] Soleja N., Mohsin M. (2024). Biotechnol. Adv..

[cit15] Zuo Y., Gou Z., Lan Y., Yan M. (2023). Trends Anal. Chem..

[cit16] Huang Y., Zhang Y., Huo F., Chao J., Cheng F., Yin C. (2020). J. Am. Chem. Soc..

[cit17] Zhang Y., Yan C., Wang C., Guo Z., Liu X., Zhu W.-H. (2020). Angew. Chem., Int. Ed..

[cit18] Liu J., Zhang W., Wang X., Ding Q., Wu C., Zhang W., Wu L., James T. D., Li P., Tang B. (2023). J. Am. Chem. Soc..

[cit19] Yue Y., Huo F., Cheng F., Zhu X., Mafireyi T., Strongin R. M., Yin C. (2019). Chem. Soc. Rev..

[cit20] Chen H., Dong B., Tang Y., Lin W. (2017). Acc. Chem. Res..

[cit21] Wang L., Du W., Hu Z., Uvdal K., Li L., Huang W. (2019). Angew. Chem., Int. Ed..

[cit22] Li J., Zhao M., Huang J., Liu P., Luo X., Zhang Y., Yan C., Zhu W.-H., Guo Z. (2022). Coord. Chem. Rev..

[cit23] Li J., Wang J., Xu L., Chi H., Liang X., Yoon J., Lin W. (2024). Angew. Chem., Int. Ed..

[cit24] Wu L., Tong Q., Cao X., Zhang D., Yang F., Lin H., Fan Q. (2025). ACS Nano.

[cit25] Ma Y., Shang J., Liu L., Li M., Xu X., Cao H., Xu L., Sun W., Song G., Zhang X.-B. (2023). J. Am. Chem. Soc..

[cit26] Shen Y., Li W., Zhou Z., Xu J., Li Y., Li H., Zheng X., Liu S., Zhang X.-B., Yuan L. (2024). Angew. Chem., Int. Ed..

[cit27] Wang X., Liew S. S., Huang J., Hu Y., Wei X., Pu K. (2024). J. Am. Chem. Soc..

[cit28] Wen Y., Hu Z., Tian W., Yan H., Huo F., Yin C. (2025). Biomaterials.

[cit29] Wu Q., Zhang Y., Jia G., Hou M., Jiang Y., Wei W., Liu P., Huang G., Zou J., Zhang J., Hai W., Zhang M., Li B., Chen X., Zhang C. (2023). Nano Today.

[cit30] Zhang X., Liu M., Hu Y., Wang X., Wei R., Yao C., Shi C., Qiu Y., Yang T., Luo X., Chen J., Sun W., Chen H., Qian X., Yang Y. (2025). Adv. Mater..

[cit31] Fan Y., Wang P., Lu Y., Wang R., Zhou L., Zheng X., Li X., Piper J. A., Zhang F. (2018). Nat. Nanotechnol..

[cit32] Schmidt E. L., Ou Z., Ximendes E., Cui H., Keck C. H. C., Jaque D., Hong G. (2024). Nat. Rev. Methods Primers.

[cit33] Lu T., Chen F. (2012). J. Comput. Chem..

[cit34] Lu T. (2024). J. Chem. Phys..

[cit35] Abbas A., Xing B., Loh T.-P. (2014). Angew. Chem., Int. Ed..

[cit36] Ariyasu S., Hayashi H., Xing B., Chiba S. (2017). Bioconjugate Chem..

[cit37] Gan H., Huang X., Luo X., Li J., Mo B., Cheng L., Shu Q., Du Z., Tang H., Sun W., Wang L., Luo S., Yu S. (2023). Adv. Healthc. Mater..

[cit38] Wang Y., Li M., Yu H., Chen Y., Cui M., Ji M., Yang F. (2024). ACS Nano.

[cit39] Badgley M. A., Kremer D. M., Maurer H. C., DelGiorno K. E., Lee H.-J., Purohit V., Sagalovskiy I. R., Ma A., Kapilian J., Firl C. E. M., Decker A. R., Sastra S. A., Palermo C. F., Andrade L. R., Sajjakulnukit P., Zhang L., Tolstyka Z. P., Hirschhorn T., Lamb C., Liu T., Gu W., Seeley E. S., Stone E., Georgiou G., Manor U., Iuga A., Wahl G. M., Stockwell B. R., Lyssiotis C. A., Olive K. P. (2020). Science.

[cit40] Qiao C., Wang L., Huang C., Jia Q., Bao W., Guo P., Tan D., Chen Z., Shi C., Rao Z., Zhang R., Wei W., Wang Z. (2025). Adv. Mater..

[cit41] Yagoda N., von Rechenberg M., Zaganjor E., Bauer A. J., Yang W. S., Fridman D. J., Wolpaw A. J., Smukste I., Peltier J. M., Boniface J. J., Smith R., Lessnick S. L., Sahasrabudhe S., Stockwell B. R. (2007). Nature.

[cit42] Ward N. P., Yoon S. J., Flynn T., Sherwood A. M., Olley M. A., Madej J., DeNicola G. M. (2024). Nat. Commun..

[cit43] Yang Y., Luo M., Zhang K., Zhang J., Gao T., Connell D. O., Yao F., Mu C., Cai B., Shang Y., Chen W. (2020). Nat. Commun..

[cit44] Yan J., Bao L., Liang H., Zhao L., Liu M., Kong L., Fan X., Liang C., Liu T., Han X., Wang K., Shen C., Sun W., Zhou X., Chu B., McGlinchey M. J., Xu X., Qiu X., Wang Y. (2025). Angew. Chem., Int. Ed..

[cit45] Jiang L., Zheng H., Lyu Q., Hayashi S., Sato K., Sekido Y., Nakamura K., Tanaka H., Ishikawa K., Kajiyama H., Mizuno M., Hori M., Toyokuni S. (2021). Redox Biol..

[cit46] Xie C., Jiang X., Yin J., Jiang R., Zhu J., Zou S. (2025). J. Hazard. Mater..

[cit47] You C., Li X., Wang D., Chen H., Liang L., Chen Y., Zhao Y., Xiang H. (2022). Angew. Chem., Int. Ed..

