# Supplementary material for: Deep-NIR to NIR-II hemicyanine fluorophore scaffolds with dual optically tunable sites for in vivo multiplexed imaging
Source: Chem Sci. 2025 Nov 3;16(48):23394–404. doi: 10.1039/d5sc06690e (PMC12598615; doi:10.1039/d5sc06690e)
Supplement: SC-016-D5SC06690E-s001 [file SC-016-D5SC06690E-s001.pdf]

Supplementary Information (SI) for Chemical Science.
This journal is © The Royal Society of Chemistry 2025

Supporting information for

Deep-NIR to NIR-II Hemicyanine Fluorophore Scaffolds with Dual Optically Tunable Sites for *in Vivo* Multiplexed Imaging

Qinian Liu, Zhuoyang Li, Yujie Huang, Zhenni Lin, Xing-Can Shen *, Hua Chen *

Key Laboratory for Chemistry and Molecular Engineering of Medicinal Resources (Ministry of Education of China), Guangxi Key Laboratory of Chemistry and Molecular Engineering of Medicinal Resources, School of Chemistry and Pharmaceutical Sciences, Guangxi Normal University, Guilin, 541004, P. R. China

*Email: chenhuagnu@gxnu.edu.cn

Table of Contents

1. Materials and instruments..3

2. Determination of the fluorescence quantum yield[1, 2]..3

3. Fluorescence and absorbance monitoring..3

4. Calculation of pK_a value 4

5. Preparation of standard solutions. 4

6. Cell culture..4

7. Cell cytotoxicity 4

8. Mitochondrial and lysosome localization exploration 5

9. Fluorescence imaging to detect Cys in PANC-1 cells and 4T1 cells under erastin induced ferroptosis model..5

10. *In vivo* imaging..5

11. Measurement of MDA, GSH and Cys in tumor tissue..6

12. DFT calculations...7

13. Ethical statement 7

14. Chemical synthesis 7

15. References 16

16. Tables and Figures..17-27

17. NMR spectra..28-39

1. Materials and instruments

Unless otherwise stated, all reagents were purchased from commercial suppliers and used without further purification. Solvents used were purified by standard methods prior to use. Twice-distilled water was used throughout all experiments. High resolution mass spectra were acquired on an LTQ FT spectrometer. ^{1}H NMR and ^{13}C NMR spectra were recorded at ambient temperature using 600 MHz spectrometer, using TMS as an internal standard. Electronic absorption spectrum was obtained on a Labtech UV Power PC spectrometer. Photoluminescent spectra were recorded at room temperature with a HITACHI F4600 fluorescence spectrophotometer with excitation and emission slit widths at 10.0 and 10.0 nm, respectively. Near-infrared second region (NIR-II) fluorescence spectra were recorded using a Horiba QM 8000 transient and steady-state fluorescence spectrometer, where the excitation and emission slit widths at 5.0 and 5.0 nm, respectively. The fluorescence imaging of cells was performed with Two-photon Confocal Scanning Laser Microscope (TCS SP8 DIVE). The pH measurements were carried out on a Mettler-Toledo Delta 320 pH meter. TLC analysis was performed on silica gel plates and column chromatography was conducted over silica gel (mesh 200–300), both of which were obtained from the Qingdao Ocean Chemicals. *In vivo* tumor imaging was performed on a Kodak *in vivo* FX Pro imaging system (Bruker), the fluorescence images were analyzed using the Bruker Molecular Imaging (BMI) Software.

2. Determination of the fluorescence quantum yield[1, 2]

Fluorescence quantum yields for **GL-1–4** were determined by using **ICG** (Φ_F = 0.13 in DMSO) as a fluorescence standard. Fluorescence quantum yields for **GL-5–8** were determined by using IR-26 (Φ_F = 0.05% in 1, 2-dichloroethane) as a fluorescence standard. The quantum yield was calculated using the following equation:

$$\Phi_{F(X)} = \Phi_{F(S)} \, (A_S F_X / A_X F_S) \, (n_X / n_S)^2$$

Where Φ_F is the fluorescence quantum yield, A is the absorbance at the excitation wavelength, F is the area under the corrected emission curve, and n is the refractive index of the solvents used. Subscripts S and X refer to the standard and to the unknown, respectively. To maximize illumination uniformity and optical transparency, the maximum absorbance of all dye solutions was maintained below 0.05. The emission spectra of the probe were obtained at its optimal excitation wavelength.

3. Fluorescence and absorbance monitoring

The fluorescence monitoring was measured using a HITACHI F4600 or Horiba QM 8000 transient and steady-state fluorescence spectrophotometer. Ultraviolet spectroscopy was carried using a Labtech UV Power PC spectrometer. The **GL-Cys** was dissolved in MeCN at a concentration of 5 mM as the stock solution, and 4 μL probe solution was transferred into a 2 mL MeCN /PBS (1:1 = v/v, pH = 7.4) quartz cuvette. After measuring the emission and absorption spectra of the test solution, the response behavior of **GL-Cys** to Cysteine (Cys) were determined by adding Cys stock solution into the test solution of **GL-Cys**.

4. Calculation of pK_a value

The pK_a value of **GL-1–6** were estimated from the changes in the fluorescence intensity with various pH values by using the Henderson Hasselbach-type mass action equation $\log [R_{max} - R) / (R - R_{min})] = pH - pK_a$. R_{min} and R_{max} are minimum and maximum limiting values of R, respectively.[3]

5. Preparation of standard solutions.

H_2O_2 stock solutions were prepared by directly diluting commercially available H_2O_2. Stock solutions of Ala, Na_2S, Met, Pro, GSH, Try, $Na_2S_2O_3$, NaCl, NaClO, $NaNO_2$, Trp, Thr, Val, $ZnCl_2$, $CaCl_2$, GSH, Hcy, Cys were purchased from different companies and prepared with distilled water. ClO^-: hypochlorite was delivered from commercial aqueous solution.

Fluorescence tests of 10 μM **GL-Cys** with various analytes under the detection conditions of Channel 1 and Channel 2：1) 10 μM **GL-Cys**; 2) 100 μM Ala; 3) 100 μM Na_2S; 4) 100 μM Met; 5) 100 μM Pro; 6) 200 μM H_2O_2; 7) 100 μM Try; 8) 100 μM $Na_2S_2O_3$; 9) 100 μM NaCl; 10) 100 μM NaClO; 11) 100 μM $NaNO_2$; 12) 100 μM Trp; 13) 100 μM Thr; 14) 100 μM Val; 15) 100 μM $ZnCl_2$; 16) 100 μM $CaCl_2$; 17) 1 mM GSH; 18) 100 μM Hcy. 19) 60 μM Cys (channel 1) / 350 μM Cys (channel 2)

6. Cell culture

PANC-1 cells and L929 cells were cultured in Dulbecco's Modified Eagle Medium (DMEM), enriched with 10% fetal bovine serum (FBS) and 1% antibiotics (consisting of 100 U/mL penicillin and 100 μg/mL streptomycin, Hyclone). The PANC-1 cells and L929 cells were maintained at 37°C in a humidified incubator with 5% CO_2 to simulate physiological conditions.

4T1 cells were cultured in 1640 medium, enriched with 10% fetal bovine serum (FBS) and 1% antibiotics (consisting of 100 U/mL penicillin and 100 μg/mL streptomycin, Hyclone). The 4T1cells were maintained at 37°C in a humidified incubator with 5% CO_2 to simulate physiological conditions.

7. Cell cytotoxicity

The *in vitro* cytotoxicity of the **GL-Cys** probe on PANC-1 cells, 4T1 cells, and L929 cells was assessed using the MTT assay. These cells were inoculated into 96-well plates and cultured for 24 h. Following this, the medium was washed three times with PBS and incubated with varying concentrations of the **GL-Cys** probe (0, 5, 10, 15, 20, and 25 μM) for 6 h. Six replicate wells were utilized for each treatment concentration. At the conclusion of the treatment, 10 μL of MTT solution (0.5 mg/mL) was added to each well, and incubation continued for an additional 4 h. Subsequently, the liquid in the plate was discarded, and 120 μL of DMSO was added and thoroughly mixed. The absorbance was measured at 570 nm using an Infinite M1000 UV-vis microplate reader (TECAN, Austria).

8. Mitochondrial and lysosome localization exploration

In the mitochondrial co-localization experiments, PANC-1 cells and 4T1 cells were seeded into a confocal dish at an appropriate density and cultured for 12 h.

Subsequently, PANC-1 cells and 4T1 cells were incubated with **GL-Cys** (10 μM) for 15 min, followed by treatment with Mito Tracker Green (Mito-Green, 500 nM) for an additional 15 min. After washing the PANC-1 cells and 4T1cellls three times with PBS, imaging was performed using a confocal laser scanning microscope at 25°C, capturing data in the green (λ_{ex} = 488 nm, λ_{em} = 500 – 550 nm) and red channels (λ_{ex} = 638 nm, λ_{em} = 660 – 780 nm).

In the lysosome co-localization experiments, PANC-1 and 4T1 cells were seeded into a confocal dish at an appropriate density and cultured for 12 h. Subsequently, PANC-1 cells and 4T1 cells were incubated with **GL-Cys** (10 μM) for 15 min, followed by treatment with Lyso-Tracker Green (Lyso-Green, 500 nM) for an additional 15 min. After washing the PANC-1 cells and 4T1cellls three times with PBS, imaging was performed using a confocal laser scanning microscope at 25°C, capturing data in the green (λ_{ex} = 488 nm, λ_{em} = 500 – 550 nm) and the red channel (λ_{ex} = 638 nm, λ_{em} = 660 – 780 nm).

9. Fluorescence imaging to detect Cys in PANC-1 cells and 4T1 cells under erastin induced ferroptosis model

The experiment involved the establishment of three sets of parallel cell samples. The first group of cells was treated with **GL-Cys** (10 μM) and co-incubated for 30 min. The second group of cells was treated with erastin (10 μM) to induce ferroptosis for 8 h, followed by the addition of **GL-Cys** (10 μM) and co-incubation for 30 min. The third group of cells was pretreated with erastin (10 μM) for 8 h, followed by treated with ferrostatin-1 (Fer-1) (10 μM) for 2 h, and then incubated with **GL-Cys** (10 μM) for 30 min. Prior to imaging, each group of cells was washed three times with PBS. All groups were observed using the red channel (λ_{ex} = 638 nm, λ_{em} = 650 – 780 nm). The pixel intensity of at least three cells under each experimental condition was quantified using ImageJ software.

10. *In vivo* imaging

All *in vivo* experiments were conducted in accordance with the guidelines established by the Guangxi Normal University Animal Study Committee regarding the care and use of laboratory animals in research. Female BALB/c nude mice (6 – 8 weeks old, weighing 20 – 22 g) were obtained from Hunan Sleek-Jinda Experimental Animal Company. The mice were randomly assigned to groups and housed under standard environmental conditions with unrestricted access to water and standard laboratory food. Prior to imaging, all mice were anesthetized using isoflurane.

PANC-1 tumor cells (1.0×10^6 cells) were resuspended in 100 μL of serum-free medium and subcutaneously injected into the flanks of each mouse. Once the tumors reached approximately 60 mm^3, the animals were subjected to the experiments. Tumor volumes were calculated using the following formula: tumor volume = (the greatest longitudinal diameter (length) × the greatest transverse diameter $(width)^2$) × 0.5. Subsequently, normal/tumor mice were divided into six groups: Group 1 (normal mice, NEM + **GL-Cys** (0.5 mM)), Group 2 (normal mice, **GL-Cys** (0.5 mM)), Group 3 (PANC-1 tumor mice, **GL-Cys** (0.5 mM)), Group 4 (PANC-1 tumor mice, NEM + **GL-Cys** (0.5 mM)), Group 5 (PANC-1 tumor mice, erastin + **GL-Cys** (0.5 mM)), and

Group 6 (PANC-1 tumor mice, erastin + ferrostatin-1 (Fer-1) + **GL-Cys** (0.5 mM)). Fluorescence imaging was performed to evaluate Cys levels *in vivo*, using channel 1 (λ_{ex} = 720 nm with a filter at λ = 790 nm) and channel 2 (λ_{ex} = 690 nm with a filter at λ = 750 nm).

4T1 tumor cells (1.0 × 10^6 cells) were resuspended in 100 μL of serum-free medium and subcutaneously injected into the flanks of each mouse. Once the tumors reached approximately 60 mm^3, the animals were subjected to the experiments. Tumor volumes were calculated using the following formula: tumor volume = (the greatest longitudinal diameter (length) × the greatest transverse diameter $(width)^2$) × 0.5. Subsequently, tumor mice were divided into four groups: Group 1 (4T1 tumor mice, **GL-Cys** (0.5 mM)), Group 2 (4T1 tumor mice, NEM + **GL-Cys** (0.5 mM)), Group 3 (4T1 tumor mice, erastin + **GL-Cys** (0.5 mM)), and Group 4 (4T1 tumor mice, erastin + Fer-1 + **GL-Cys** (0.5 mM)). Fluorescence imaging was performed to evaluate Cys levels *in vivo*, using channel 1 (λ_{ex} = 720 nm with a filter at λ = 790 nm) and channel 2 (λ_{ex} = 690 nm with a filter at λ = 750 nm). All results are expressed as the mean ± SD (n = 3), and images were analyzed using Bruker Molecular Imaging (BMI) Software.

11. Measurement of MDA, GSH and Cys in tumor tissue

The PANC-1 tumor tissue and tumor ferroptosis tissue were dissected and stored at 0°C for subsequent analysis. The levels of malondialdehyde (MDA), glutathione (GSH), and Cys were quantified using assay kits from Beijing Solarbio Science & Technology Co., Ltd., following the manufacturer's instructions. MDA, an indicator of lipid peroxidation, reacts with thiobarbituric acid to form a brownish-red compound, 3,5,5-trimethyloxazole-2,4-dione, which has a maximum absorbance at 532 nm. The GSH assay utilizes a GSH recycling system, incorporating GSH substrate (DTNB) and GSH reductase. The reaction between DTNB and GSH produces 2-nitro-5-mercaptobenzoic acid, which is characterized by a yellow color. Therefore, the concentration of GSH was determined by measuring the absorbance at 412 nm. The Cys assay is based on the reduction of phosphotungstic acid by Cys, resulting in the formation of tungsten blue, which exhibits a blue color. Consequently, the concentration of Cys was assessed by measuring the absorbance at 600 nm. Absorbance readings for MDA, GSH, and Cys were obtained using a spectrophotometer set to 532 nm, 412 nm, and 600 nm, respectively.

The 4T1 tumor tissue and tumor ferroptosis tissue were dissected and stored at 0°C for subsequent analysis. The levels of malondialdehyde (MDA), glutathione (GSH), and Cys were quantified using assay kits from Beijing Solarbio Science & Technology Co., Ltd., following the manufacturer's instructions. MDA, an indicator of lipid peroxidation, reacts with thiobarbituric acid to form a brownish-red compound, 3,5,5-trimethyloxazole-2,4-dione, which has a maximum absorbance at 532 nm. The GSH assay utilizes a GSH recycling system, incorporating GSH substrate (DTNB) and GSH reductase. The reaction between DTNB and GSH produces 2-nitro-5-mercaptobenzoic acid, which is characterized by a yellow color. Therefore, the concentration of GSH was determined by measuring the absorbance at 412 nm. The Cys assay is based on the reduction of phosphotungstic acid by Cys, resulting in the formation of tungsten blue, which exhibits a blue color. Consequently, the concentration of Cys was assessed by

measuring the absorbance at 600 nm. Absorbance readings for MDA, GSH, and Cys were obtained using a spectrophotometer set to 532 nm, 412 nm, and 600 nm, respectively.

12. DFT calculations

All the calculations were carried out using the Gaussian 09 program and further processed employing the Multiwfn and VMD programs simultaneously. All the geometries of **GL-1–8**, **GL-OH, GL-Ac, GL-OH-Cys**, and **GL-Cys** were optimized at 6-31G(d) level.

13. Ethical statement

The authors declare that all animal experiments were carried out according to the guidelines approved by the Guangxi Normal University Animal Study Committee for the care and use of laboratory animals in research (No. 202405-001). All authors comply with all relevant ethical regulations.

14. Chemical synthesis

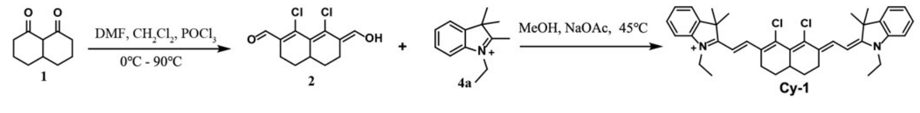

Scheme S1. Synthetic route for compound **Cy-1**.

Synthesis of compound Cy-1.[4] To a reaction flask, anhydrous dimethylformamide (DMF, 5 mL) and anhydrous dichloromethane (CH_2Cl_2, 5 mL) were introduced. The flask was subsequently sealed under an inert nitrogen atmosphere and cooled to 0°C. Phosphorus oxychloride ($POCl_3$, 3 mL) was then added dropwise to the cooled solution, and the mixture was stirred at 0°C for 30 min. Following this, decahydronaphthalene-1,8-dione (500.0 mg, 3.00 mmol) was added to the reaction vessel. The reaction mixture was heated to reflux at 90°C under a nitrogen atmosphere with continuous stirring for 4 h. Upon completion, the mixture was quenched by pouring onto crushed ice, and the resultant product was extracted with dichloromethane (5 x 100 mL). The combined organic extracts were dried over anhydrous sodium sulfate (Na_2SO_4) and concentrated under reduced pressure to afford compound **2** as an orange viscous liquid (450.0 mg, 58.0% yield), which was utilized in subsequent reactions without further purification.

In a separate procedure, compound **2** (200.0 mg, 0.77 mmol), compound **4a** (430.0 mg, 2.31 mmol), and sodium acetate (51.0 mg, 0.77 mmol) were combined in a flask containing methanol (MeOH, 5 mL). The mixture was stirred at 45°C under a nitrogen atmosphere for 4 h. After the reaction period, the mixture was cooled to ambient temperature. The solvent was then removed under reduced pressure using a rotary evaporator to yield a crude product. The crude product was purified by silica gel column chromatography, employing a gradient elution of dichloromethane to methanol (100:1 to 50:1, v/v). The desired product was isolated as a dark-green solid (175.0 mg, 0.29 mmol, 37.7% yield). ^1H NMR (600 MHz, Methanol-d_4) δ 8.37 (d, J = 14.2 Hz, 2H), 7.53 (d, J = 7.9 Hz, 2H), 7.45 (d, J = 6.6 Hz, 2H), 7.34 (d, J = 7.9 Hz, 2H), 7.30 (t, J = 7.4 Hz, 2H), 6.34 (d, J = 14.2 Hz, 2H), 4.26 – 4.22 (m, 4H), 2.25 – 2.17 (m, 4H), 1.78

(s, 1H), 1.73 (s, 12H), 1.59 (t, $J = 7.4$ Hz, 4H), 1.43 (d, $J = 7.2$ Hz, 6H). ^{13}C NMR (150 MHz, Methanol-d_4) δ 172.84, 151.47, 145.34, 144.36, 143.37, 143.10, 142.84, 142.01, 141.91, 140.07, 136.28, 133.37, 131.82, 131.42, 130.86, 129.94, 126.51, 124.31, 123.58, 116.32, 114.97, 112.00, 103.25, 54.81, 50.62, 40.58, 40.21, 39.15, 30.75, 30.65, 28.08, 27.16, 27.04, 26.88, 26.79, 23.74, 23.03, 12.60. HRMS (ESI) m/z Calcd for $C_{38}H_{43}Cl_2N_2^+$ ([M]$^+$): 597.2798; found: 597.2781.

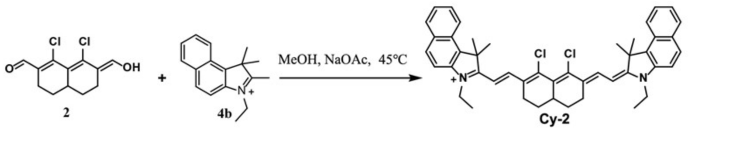

Scheme S2. Synthetic route for compound **Cy-2**.

Synthesis of compound Cy-2. Compound **2** (200.0 mg, 0.77 mmol), compound **4b** (430.0 mg, 2.31 mmol) and sodium acetate (51.0 mg, 0.77 mmol) were mixed in a flask containing MeOH (5 mL), and the mixture was stirred at 45°C under nitrogen atmosphere for 4 h. After the reaction period, the mixture was cooled to ambient temperature. The resulting reaction mixture was then obtained through spin drying using a vacuum evaporator. The dark-green solid was purified by silica column chromatography using CH_2Cl_2/MeOH (100:1 to 50:1). The desired product was obtained as a dark-green solid (130.0 mg, 0.24mmol, 24.3%). ^{1}H NMR (600 MHz, Methanol-d_4) δ 7.98 (d, $J = 14.1$ Hz, 2H), 7.52 (d, $J = 8.4$ Hz, 2H), 7.29 (d, $J = 8.5$ Hz, 2H), 7.21 – 7.18 (m, 3H), 7.11 – 7.07 (m, 3H), 6.45 (d, $J = 8.7$ Hz, 2H), 5.47 (d, $J =$ 14.2 Hz, 2H), 4.76 (s, 2H), 4.58 (d, $J = 9.7$ Hz, 2H), 1.77 (t, $J = 6.1$ Hz, 4H), 1.46 (t, J = 7.6 Hz, 3H), 1.35 (s, 12H), 1.28 – 1.21 (m, 8H). ^{13}C NMR (150 MHz, $CDCl_3$-d) δ 175.94, 175.01, 149.28, 147.77, 147.21, 146.16, 142.44, 142.17, 139.14, 139.03, 134.48, 132.17, 130.96, 129.94, 129.64, 129.54, 128.32, 128.08, 127.43, 127.17, 125.45, 124.60, 124.50, 124.11, 123.61, 123.55, 122.62, 121.94, 118.99, 114.20, 103.69, 53.58, 51.63, 49.68, 40.18, 29.32, 29.08, 28.15, 28.10, 27.34, 25.67, 24.27, 22.82, 20.75, 14.26, 12.77. HRMS (ESI) Calcd for $C_{46}H_{47}Cl_2N_2^+$ ([M]$^+$): 697.3111; found: 697.3131.

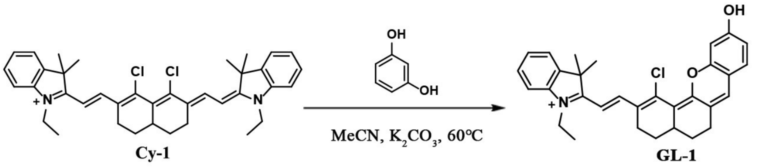

Scheme S3. Synthetic route for compound **GL-1**.

Synthesis of compound GL-1. Compound **Cy-1** (100.1 mg, 0.16 mmol), resorcinol (160.0 mg, 1.45 mmol), and K_2CO_3 (10.0 mg, 0.07 mmol) were dissolved in 4 mL of acetonitrile (MeCN). The solution was heated to 60°C and allowed to react for 2 h. Upon completion of the reaction, the mixture was cooled to room temperature and poured into approximately 100.1 g of ice water. Subsequently, perchloric acid (70%; 2 mL) was added, followed by extraction with dichloromethane. The water was removed using anhydrous sodium sulfate. The mixture was then concentrated under reduced

pressure to yield a crude black solid. The crude product was purified via silica gel column chromatography, employing a CH_2Cl_2/MeOH gradient eluent (ranging from 80:1 to 80:1.5). The target product was ultimately obtained as a green solid (27.0 mg, 0.20 mmol, yield 32.9%). ^{1}H NMR (600 MHz, Methanol-d_4) δ 7.72 (d, J = 2.4 Hz, 1H), 7.67 – 7.65 (m, 1H), 7.58 – 7.52 (m, 2H), 7.45 (dd, J = 7.3, 1.2 Hz, 1H), 7.32 (d, J = 8.5 Hz, 1H), 7.22 (s, 1H), 6.77 (dd, J = 8.5, 2.3 Hz, 1H), 6.74 (d, J = 2.3 Hz, 1H), 6.58 (d, J = 14.8 Hz, 1H), 4.41 (q, J = 7.4 Hz, 2H), 3.23 – 3.20 (m, 1H), 2.72 – 2.58 (m, 4H), 1.79 (s, 3H), 1.78 (s, 3H), 1.71 – 1.66 (m, 4H), 1.48 (s, 3H). ^{13}C NMR (150 MHz, Methanol-d_4) δ 178.21, 169.33, 158.85, 149.71, 146.55, 143.84, 142.58, 133.61, 132.40, 130.85, 130.68, 129.87, 129.64, 128.59, 126.26, 125.49, 124.69, 123.89, 116.00, 113.79, 103.06, 41.62, 40.18, 33.08, 29.72, 29.16, 28.12, 27.85, 27.42, 26.93, 14.41. HRMS (ESI) Calcd for $C_{31}H_{31}ClNO_2^+$ ([M]$^+$): 484.2038; found: 484.2039.

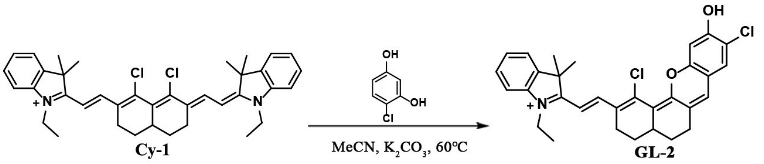

Scheme S4. Synthetic route for compound **GL-2**.

Synthesis of compound GL-2. Compound **Cy-1** (100.0 mg, 0.16 mmol), 4-chlororesorcinol (210.0 mg, 1.46 mmol), and K_2CO_3 (10.0 mg, 0.07 mmol) were dissolved in 4 mL of MeCN. The solution was heated to 60°C and allowed to react for 2 h. Upon completion of the reaction, the mixture was cooled to room temperature and poured into approximately 100.0 g of ice water. Subsequently, perchloric acid (70%; 2 mL) was added, followed by extraction with dichloromethane. The water was removed using anhydrous sodium sulfate. The mixture was then concentrated under reduced pressure to yield a crude black solid. The crude product was purified via silica gel column chromatography, employing a CH_2Cl_2/MeOH gradient eluent (ranging from 80:1 to 80:1.5). The target product was ultimately obtained as a green solid (25.0 mg, 0.20 mmol, yield 28.7%). ^{1}H NMR (600 MHz, DMSO-d_6) δ 7.74 (s, 1H), 7.56 (d, J = 5.3 Hz, 1H), 7.43 – 7.38 (m, 1H), 7.32 (s, 1H), 7.27 (t, J = 7.7 Hz, 1H), 7.07 (d, J = 7.9 Hz, 1H), 7.03 (d, J = 7.4 Hz, 1H), 6.67 (s, 1H), 6.36 (s, 1H), 4.00 – 3.89 (m, 2H), 2.96 – 2.86 (m, 1H), 2.72 – 2.66 (m, 2H), 2.66 (s, 2H), 1.67 (s, 5H), 1.60 (s, 7H), 1.34 (t, J = 7.2 Hz, 2H). ^{13}C NMR (150 MHz, DMSO-d_6) δ 174.35, 165.28,155.69, 142.84, 142.00, 141.09, 139.57, 133.51, 131.30, 129.59, 128.39, 128.16, 127.64, 124.11, 122.38, 121.98, 120.93, 119.79, 116.76,114.76, 102.48, 46.91, 38.49, 31.30, 30.38, 29.04, 27.79, 26.98, 25.40, 22.10, 11.42. HRMS (ESI) Calcd for $C_{31}H_{30}Cl_2NO_2^+$ ([M]$^+$): 518.1648; found: 518.1647.

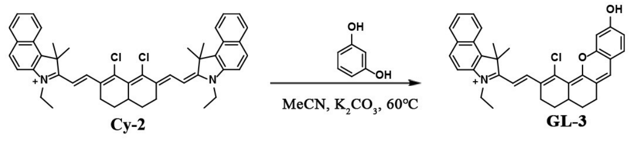

Scheme S5. Synthetic route for compound **GL-3**.

Synthesis of compound GL-3. Compound **Cy-2** (100.0 mg, 0.14 mmol), resorcinol (160.0 mg, 1.45 mmol), and K_2CO_3 (10.0 mg, 0.07 mmol) were dissolved in 4 mL of MeCN. The solution was heated to 60°C and allowed to react for 2 h. Upon completion of the reaction, the mixture was cooled to room temperature and poured into approximately 100.0 g of ice water. Subsequently, perchloric acid (70%; 2 mL) was added, followed by extraction with dichloromethane. The water was removed using anhydrous sodium sulfate. The mixture was then concentrated under reduced pressure to yield a crude black solid. The crude product was purified via silica gel column chromatography, employing a CH_2Cl_2/MeOH gradient eluent (ranging from 80:1 to 80:1.5). The target product was ultimately obtained as a green solid (23.0 mg, 0.04 mmol, yield 30.0%). ^{1}H NMR (600 MHz, DMSO-d_6) δ 8.68 (d, J = 15.2 Hz, 1H), 8.38 (d, J = 9.3 Hz, 1H), 8.23 (d, J = 8.9 Hz, 1H), 8.17 (d, J = 8.3 Hz, 1H), 8.00 (d, J = 8.9 Hz, 1H), 7.76 – 7.71 (m, 1H), 7.33 (d, J = 8.4 Hz, 1H), 7.15 (d, J = 8.7 Hz, 1H), 6.79 (s, 1H), 6.77 – 6.73 (m, 1H), 6.72 (d, J = 2.3 Hz, 1H), 4.67 – 4.61 (m, 2H), 2.95 – 2.92 (m, 1H), 2.72 – 2.50 (m, 2H), 1.98 (s, 9H), 1.96 (s, 4H), 1.46 (d, J = 7.2 Hz, 3H). ^{13}C NMR (150 MHz, DMSO-d_6) δ 177.95, 164.66, 158.20, 153.35, 146.56, 142.70, 142.20, 138.50, 136.84, 133.41, 132.93, 132.53, 129.55, 129.39, 128.92, 128.25, 128.16, 127.46, 125.85, 122.70, 121.12, 120.97, 113.66, 113.45, 102.37, 45.76, 41.07, 33.56, 29.32, 28.98, 28.93, 28.60, 28.01, 26.41, 13.14. HRMS (ESI) Calcd for $C_{35}H_{33}ClNO_2^+$ ($[M]^+$): 534.2194; found: 534.2194.

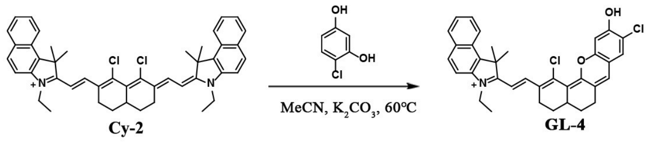

Scheme S6. Synthetic route for compound **GL-4**.

Synthesis of compound GL-4. Compound **Cy-2** (100.0 mg, 0.14 mmol), 4-chlororesorcinol (210.0 mg, 1.46 mmol), and K_2CO_3 (10.0 mg, 0.07 mmol) were dissolved in 4 mL of acetonitrile (MeCN). The solution was heated to 60°C and allowed to react for 2 h. Upon completion of the reaction, the mixture was cooled to room temperature and poured into approximately 100.0 g of ice water. Subsequently, perchloric acid (70%; 2 mL) was added, followed by extraction with dichloromethane. The water was removed using anhydrous sodium sulfate. The mixture was then concentrated under reduced pressure to yield a crude black solid. The crude product was purified via silica gel column chromatography, employing a CH_2Cl_2/MeOH gradient eluent (ranging from 80:1 to 80:1.5). The target product was ultimately obtained as a green solid (20.0 mg, 0.04 mmol, yield 25.0%). ^{1}H NMR (600 MHz, DMSO-d_6) δ 8.16 (d, J = 8.5 Hz, 1H), 8.07 (d, J = 12.9 Hz, 1H), 7.94 (s, 1H), 7.72 (s, 1H), 7.52 (d, J = 9.0 Hz, 2H), 7.37 (t, J = 7.4 Hz, 1H), 7.26 (d, J = 8.8 Hz, 1H), 7.16 (d, J = 8.2 Hz, 1H), 6.41 (s, 1H), 5.76 (s, 1H), 4.10 (m, 2H), 2.96 – 2.92 (m, 1H), 2.73 – 2.65 (m, 2H), 2.61 (t, J = 11.9 Hz, 2H), 2.10 – 1.95 (m, 4H), 1.90 (s, 6H), 1.35 (s, 3H). ^{13}C NMR (150 MHz, DMSO-d_6) δ 172.34, 164.75, 154.93, 152.16, 150.03, 141.64, 140.39, 133.06, 131.28, 130.38, 130.01, 129.80, 129.58, 128.03, 127.58, 127.31, 126.75, 126.46, 124.11, 123.51, 121.79, 117.08, 116.49, 110.58, 102.54, 54.93, 47.05,

34.43, 31.29, 30.38, 29.42, 29.09, 28.71, 26.82, 13.95. HRMS (ESI) Calcd for $C_{35}H_{32}Cl_2NO_2^+$ ([M]$^+$): 568.1805; found: 568.1805.

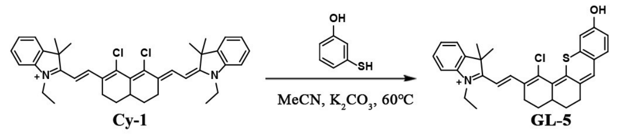

Scheme S7. Synthetic route for compound **GL-5**.

Synthesis of compound GL-5. Compound **Cy-1** (40.0 mg, 0.06 mmol), 3-hydroxybenzenethiol (4.0 mg, 0.03 mmol), and K_2CO_3 (10.0 mg, 0.07 mmol) were dissolved in 6 mL of MeCN. The solution was heated to 60°C and reacted for 2 h. After the reaction was complete, the mixture was cooled to room temperature and poured into approximately 100.0 g of ice water. Subsequently, perchloric acid (70%; 2 mL) was added, followed by extraction with dichloromethane. Anhydrous sodium sulfate was used to remove moisture. The mixture was then concentrated under reduced pressure to obtain a crude black solid. The crude product was purified by silica gel column chromatography using a CH_2Cl_2/MeOH gradient elution (from a ratio of 80:1 to 80:1.5). The final target product obtained was a green solid (10.0 mg, 0.02 mmol, yield 27.2%). ^{1}H NMR (600 MHz, DMSO-d_6) δ 7.96 (d, J = 9.0 Hz, 1H), 7.84 (d, J = 2.5 Hz, 1H), 7.63 (dd, J = 6.5, 2.9 Hz, 2H), 7.60 (d, J = 7.7 Hz, 1H), 7.57 (d, J = 6.8 Hz, 1H), 7.36 (d, J = 8.5 Hz, 1H), 7.02 (s, 1H), 6.86 (d, J = 2.7 Hz, 1H), 6.80 (dd, J = 8.5, 2.4 Hz, 1H), 4.49 (q, J = 7.3 Hz, 2H), 2.71 – 2.65 (m, 3H), 2.63 – 2.58 (m, 2H), 1.73 (d, J = 7.5 Hz, 6H), 1.42 (dd, J = 16.8, 7.3 Hz, 7H). ^{13}C NMR (150 MHz, DMSO-d_6) δ 167.99, 158.43, 148.46, 143.17, 141.98, 140.82, 135.24, 132.01, 131.83, 131.40, 131.24, 129.42, 128.99, 128.60, 126.86, 123.56, 123.08, 120.21, 118.11, 114.36, 110.07, 43.00, 40.52, 31.93, 29.71, 28.97, 28.88, 26.76, 26.57, 26.40, 13.24. $C_{31}H_{31}ClNOS^+$ ([M]$^+$): 500.1810; found: 500.1800.

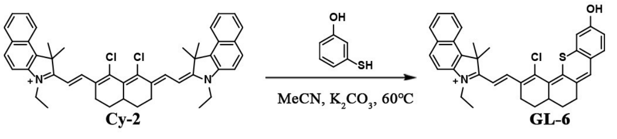

Scheme S8. Synthetic route for compound **GL-6**.

Synthesis of compound GL-6. Compound **Cy-2** (40.0 mg, 0.05 mmol), 3-hydroxythiophenol (4.0 mg, 0.04 mmol), and K_2CO_3 (10.0 mg, 0.07 mmol) were dissolved in 6 mL of MeCN. The solution was heated to 60°C and allowed to react for 2 h. Upon completion of the reaction, the mixture was cooled to room temperature and poured into approximately 100.0 g of ice water. Subsequently, perchloric acid (70%; 2 mL) was added, followed by extraction with dichloromethane. The water was removed using anhydrous sodium sulfate. The mixture was then concentrated under reduced pressure to yield a crude black solid. The crude product was purified via silica gel column chromatography, employing a CH_2Cl_2/MeOH gradient eluent (ranging from 80:1 to 80:1.5). The target product was ultimately obtained as a green solid (7.8 mg,

0.01 mmol, yield 24.3%). ^{1}H NMR (150 MHz, DMSO-d_6) δ 8.58 (d, J = 15.4 Hz, 1H), 8.41 (s, 1H), 8.37 (d, J = 8.4 Hz, 1H), 8.26 (s, 1H), 8.21 (d, J = 4.0 Hz, 1H), 8.16 (s, 1H), 8.09 (d, J = 3.7 Hz, 1H), 7.34 (s, 1H), 6.99 (s, 1H), 6.94 (d, J = 4.5 Hz, 1H), 6.86 (d, J = 2.4 Hz, 1H), 6.79 (dd, J = 8.5, 2.3 Hz, 1H), 4.62 (q, J = 7.3 Hz, 2H), 2.77 – 2.64 (m, 4H), 2.61 (t, J = 4.5 Hz, 1H), 2.18 – 2.13 (m, 2H), 2.12 – 2.08 (m, 2H), 1.76 (s, 6H), 1.16 (t, J = 8.1 Hz, 3H). ^{13}C NMR (150 MHz, DMSO-d_6) δ 179.49, 147.35, 141.34, 138.52, 138.21, 137.75, 137.05, 135.14, 133.05, 131.94, 130.73, 130.07, 129.74, 128.42, 127.29, 127.26, 126.93, 126.46, 123.41, 123.00, 121.07, 120.20, 113.17, 112.88, 109.11, 55.45, 52.93, 43.30, 31.89, 29.41, 26.49, 26.26, 21.46, 13.56, 12.86. $C_{35}H_{33}ClNOS^+$ ([M]$^+$): 550.1966; found: 550.1950.

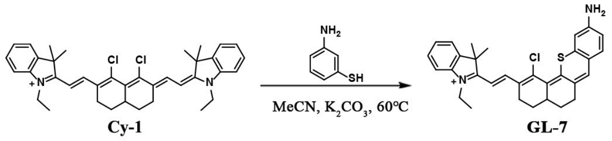

Scheme S9. Synthetic route for compound **GL-7**.

Synthesis of compound GL-7. Compound **Cy-1** (40.0 mg, 0.06 mmol), 3-aminothiophenol (4.0 mg, 0.03 mmol), and K_2CO_3 (10 mg, 0.07 mmol) were dissolved in 6 mL of MeCN. The solution was heated to 60°C and allowed to react for 2 h. Upon completion of the reaction, the mixture was cooled to room temperature and poured into approximately 100.0 g of ice water. Subsequently, perchloric acid (70%; 2 mL) was added, followed by extraction with dichloromethane. The water was removed using anhydrous sodium sulfate. The mixture was then concentrated under reduced pressure to yield a crude black solid. The crude product was purified via silica gel column chromatography, employing a CH_2Cl_2/MeOH gradient eluent (ranging from 80:1 to 80:1.5). The target product was ultimately obtained as a green solid (10.2 mg, 0.01 mmol, yield 29.0%). ^{1}H NMR (600 MHz, DMSO-d_6) δ 8.49 (d, J = 15.3 Hz, 1H), 7.91 – 7.83 (m, 2H), 7.61 (p, J = 6.7, 5.4 Hz, 2H), 7.56 (t, J = 7.3 Hz, 1H), 7.36 (d, J = 8.4 Hz, 1H), 7.03 (s, 1H), 6.87 (d, J = 16.3 Hz, 2H), 4.64 – 4.53 (m, 2H), 2.89 (d, J = 17.4 Hz, 1H), 2.76 – 2.56 (m, 4H), 2.06 (dd, J = 94.8, 21.9 Hz, 4H), 1.73 (d, J = 7.9 Hz, 6H), 1.42 (d, J = 6.9 Hz, 3H). ^{13}C NMR (150 MHz, DMSO-d_6) δ 178.54, 158.43, 148.44, 143.16, 141.92, 141.52, 140.82, 135.23, 131.99, 131.81, 131.38, 131.24, 129.65, 129.19, 128.59, 126.84, 123.08, 120.19, 114.36, 110.07, 109.95, 54.93, 51.29, 29.08, 29.03, 28.96, 28.86, 26.75, 26.56, 26.41, 13.23. $C_{31}H_{32}ClN_2S^+$ ([M]$^+$): 499.1970; found: 499.1964.

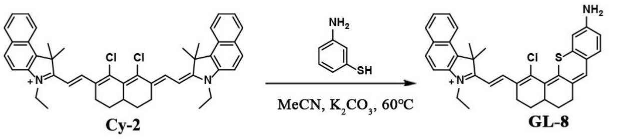

Scheme S10. Synthetic route for compound **GL-8**.

Synthesis of compound GL-8. Compound **Cy-2** (40.0 mg, 0.05 mmol), 3-aminothiophenol (4.0 mg, 0.03 mmol), and K_2CO_3 (10.0 mg, 0.07 mmol) were dissolved in 6 mL of MeCN. The solution was heated to 60°C and allowed to react for

2 h. Upon completion of the reaction, the mixture was cooled to room temperature and poured into approximately 100.0 g of ice water. Subsequently, perchloric acid (70%; 2 mL) was added, followed by extraction with dichloromethane. The water was removed using anhydrous sodium sulfate. The mixture was then concentrated under reduced pressure to yield a crude black solid. The crude product was purified via silica gel column chromatography, employing a CH_2Cl_2/MeOH gradient eluent (ranging from 80:1 to 80:1.5). The target product was ultimately obtained as a green solid (8.3 mg, 0.01 mmol, yield 25.6%). ^{1}H NMR (600 MHz, DMSO-d_6) δ 8.47 (d, J = 14.7 Hz, 1H), 8.35 (d, J = 8.5 Hz, 1H), 8.18 (d, J = 8.9 Hz, 1H), 8.13 (d, J = 7.2 Hz, 1H), 7.92 (d, J = 9.0 Hz, 1H), 7.72 (dd, J = 18.2, 8.3 Hz, 2H), 7.62 – 7.59 (m, 1H), 7.38 (d, J = 9.2 Hz, 1H), 6.75 (d, J = 7.1 Hz, 2H), 6.60 (d, J = 14.8 Hz, 1H), 4.57 – 4.50 (m, 2H), 2.73 – 2.70 (m, 1H), 2.70 – 2.51 (m, 4H), 2.14 – 2.06 (m, 2H), 1.95 (d, J = 8.6 Hz, 6H), 1.93 – 1.83 (m, 2H), 1.42 (t, J = 7.2 Hz, 3H). ^{13}C NMR (150 MHz, DMSO-d_6) δ 176.00, 151.42, 146.05, 144.89, 141.99, 138.96, 137.89, 136.04, 135.69, 132.14, 130.93, 130.73, 130.01, 128.09, 127.23, 126.46, 125.94, 123.41, 122.61, 121.22, 117.97, 116.17, 112.20, 106.18, 105.11, 51.77, 45.45, 31.99, 29.41, 29.02, 26.82, 26.61, 26.36, 21.46, 12.97. $C_{35}H_{34}ClN_2S^+$ ([M]$^+$): 549.2126; found: 549.2111.

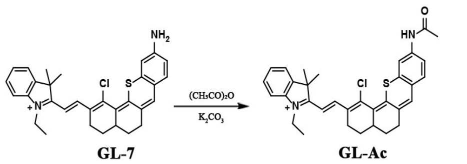

Scheme S11. Synthetic route for compound **GL-Ac**.

Synthesis of compound GL-Ac. Compound **GL-7** (40.0 mg, 0.06 mmol) and K_2CO_3 (10.0 mg, 0.07 mmol) were dissolved in MeCN (4 mL), and acetic anhydride (2 mL) was added. The reaction mixture was stirred at room temperature for 6 h. After completion, the solvent was removed under reduced pressure to yield a crude dark green solid. Purification by flash column chromatography on silica gel (gradient elution: CH_2Cl_2/MeOH, 80:1 to 80:1.5) afforded the desired product as a dark green solid (31.4 mg, 0.01 mmol, yield 73.6%).^{1}H NMR (600 MHz, DMSO-d_6) δ 7.65 (d, J = 15.4 Hz, 1H), 7.11 (s, 1H), 7.03 (dd, J = 11.8, 7.6 Hz, 2H), 6.79 – 6.73 (m, 2H), 6.56 (d, J = 8.5 Hz, 1H), 6.48 (d, J = 8.4 Hz, 1H), 6.14 – 6.07 (m, 2H), 2.07 – 2.03 (m, 1H), 1.90 – 1.73 (m, 4H), 1.66 (s, 5H), 1.33 – 1.26 (m, 2H), 1.24 (s, 3H), 1.15 (d, J = 24.4 Hz, 2H), 0.90 (d, J = 8.5 Hz, 6H). ^{13}C NMR (150 MHz, DMSO-d_6) δ 178.99, 168.95, 148.56, 143.30, 141.48, 140.74, 140.06, 139.78, 133.99, 133.65, 132.50, 129.99, 129.64, 129.22, 128.85, 127.56, 123.22, 123.12, 117.96, 114.60, 113.06, 110.96, 54.93, 51.48, 31.89, 31.28, 29.79, 28.84, 26.65, 26.47, 24.18, 22.09, 13.34. HRMS (ESI) Calcd for $C_{33}H_{34}ClN_2OS^+$ ([M]$^+$):541.2075, found: 541.2009.

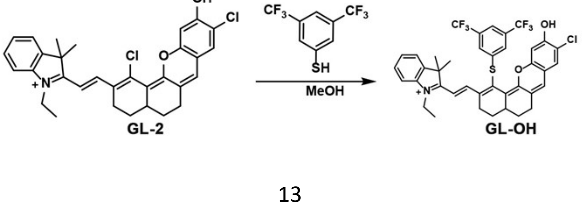

Scheme S12. Synthetic route for compound **GL-OH**.

Synthesis of compound GL-OH. Compound **GL-2** (50.0 mg, 0.09 mmol), 3,5-bis(trifluoromethyl)benzenethiol (29.4 mg, 0.12 mmol) were dissolved in MeOH (4 mL), raise the temperature to 40°C for reaction 4 h, after the reaction is completed, it is dried by rotary evaporation. The resulting green crude product was purified by silica gel column chromatography using CH_2Cl_2/MeOH (with a gradient elution from 80:1 to 50:1) as the eluent, yielding a green solid. The target product was obtained (31.0 mg, 0.04 mmol) with a yield of 62.0%. ^{1}H NMR (600 MHz, DMSO-d_6) δ 8.06 (s, 1H), 7.87 (s, 1H), 7.77 (s, 1H), 7.64 (s, 1H), 7.55 (d, J = 5.5 Hz, 1H), 7.36 (d, J = 7.2 Hz, 1H), 7.25 (t, J = 7.6 Hz, 2H), 7.07 (s, 1H), 7.03 – 6.97 (m, 1H), 6.89 (s, 1H), 6.69 (d, J = 43.9 Hz, 1H), 4.05 – 3.87 (m, 2H), 3.02 – 2.96 (m, 1H), 2.72 (td, J = 33.6, 33.2, 21.0 Hz, 4H), 2.20 – 2.09 (m, 2H), 2.05 – 1.94 (m, 3H), 1.45 (s, 6H), 1.34 (s, 3H). ^{13}C NMR (150 MHz, DMSO-d_6) δ 174.30, 154.94, 152.28, 148.63, 142.65, 141.80, 139.13, 133.51, 133.16, 133.09, 131.54, 130.88, 130.67, 130.32, 129.63, 129.58, 128.18, 127.74, 127.61, 126.91, 126.88, 125.63, 124.10, 123.82, 122.01, 120.20, 118.71, 117.16, 102.24, 37.80, 35.11, 33.68, 31.28, 30.38, 29.03, 26.55, 25.12, 22.09, 13.94. HRMS (ESI) Calcd for $C_{39}H_{33}ClF_6NO_2S^+$ ($[M]^+$):728.1819, found: 728.1815.

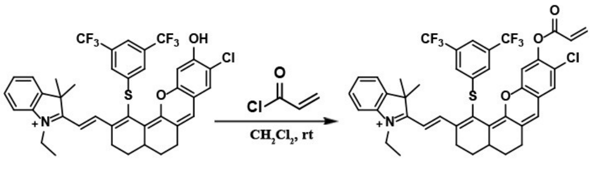

Scheme S13. Synthetic route for compound **GL-Cys**.

Synthesis of compound GL-Cys. Compound **GL-OH** (50.0 mg, 0.07 mmol) was dissolved in CH_2Cl_2 (10 mL), followed by the addition of acryloyl chloride (41.4 mg, 0.46 mmol). The reaction mixture was stirred at room temperature under a nitrogen atmosphere for 3 h. After completion, the mixture was extracted using a separatory funnel and concentrated under reduced pressure to obtain the crude product. Purification was performed via silica gel column chromatography with CH_2Cl_2/MeOH (80:1 to 50:1) as the eluent, affording the blue solid **GL-Cys** (39.8 mg, 0.05 mmol) in a yield of 73.0%. ^{1}H NMR (600 MHz, DMSO-d_6) δ 7.90 (t, J = 16.9 Hz, 2H), 7.79 (d, J = 17.6 Hz, 2H), 7.68 (d, J = 17.8 Hz, 2H), 7.58 (d, J = 17.5 Hz, 2H), 7.45 (s, 1H), 7.23 (d, J = 42.1 Hz, 1H), 7.04 (d, J = 15.0 Hz, 1H), 6.89 (d, J = 15.3 Hz, 1H), 6.77 – 6.53 (m, 2H), 6.22 (d, J = 40.9 Hz, 1H), 4.58 (d, J = 37.5 Hz, 2H), 3.05 (d, J = 18.0 Hz, 1H), 2.83 – 2.66 (m, 2H), 2.29 – 2.16 (m, 2H), 1.97 (d, J = 21.5 Hz, 2H), 1.63 (d, J = 25.3 Hz, 6H), 1.41 (s, 3H), 1.33 (s, 2H). ^{13}C NMR (150 MHz, DMSO-d_6) δ 166.73, 162.48, 149.93, 148.64, 146.34, 143.08, 142.77, 140.48, 134.89, 132.00, 131.45, 130.42, 130.18, 129.42, 129.05, 128.97, 128.85, 128.47, 128.30, 126.75, 126.53, 126.45, 125.25, 123.43, 122.92, 122.85, 121.62, 120.51, 119.80, 119.54, 114.60, 102.51, 41.66, 41.17, 37.83, 29.57, 28.81, 28.14, 27.78, 27.60, 26.23, 13.71. HRMS (ESI) Calcd for $C_{42}H_{35}ClF_6NO_3S^+$ ($[M]^+$):782.1925, found:782.1960.

15. References

(1) Swamy, M. M. M.; Murai, Y.; Monde, K.; Tsuboi, S.; Swamy, A. K.; Jin, T. Biocompatible and water-soluble shortwave-infrared (SWIR)-emitting cyanine-based fluorescent probes for *in vivo* multiplexed molecular imaging. *ACS Appl. Mater. Interfaces* **2024**, *16* (14), 17253-17266.
(2) Ou, Y.-F.; Xiang, H.-Y.; Yang, X.; Wang, R.-X.; Huan, S.-Y.; Yuan, L.; Ren, T.-B.; Zhang, X.-B. Constructing stable and wavelength-extended heptamethine cyanines via donor ectopic substitution for NIR-IIa/b bioimaging. *Angew. Chem. Int. Ed.* **2025**, e202423978.
(3) Wang, L.; He, M.; Liu, X.; Jiang, B.-P.; Chen, H.; Shen, X.-C. Dual-labeled single fluorescent probes for the simultaneous two-color visualization of dual organelles and for monitoring cell autophagy. *Anal. Chem.* **2024**, *96* (2), 876-886.
(4) Hu, B.; Liu, Q.; Jiang, Y.; Huang, Y.; Ji, H.; Zhang, J.; Wang, X.; Shen, X.-C.; Chen, H. NIR-II fluorescence/photoacoustic dual ratiometric probes with unique recognition site for quantitatively visualizing H_2S_2 *in vivo*. *Angew. Chem. Int. Ed.* **2024**, e202418378.

16. Tables and Figures

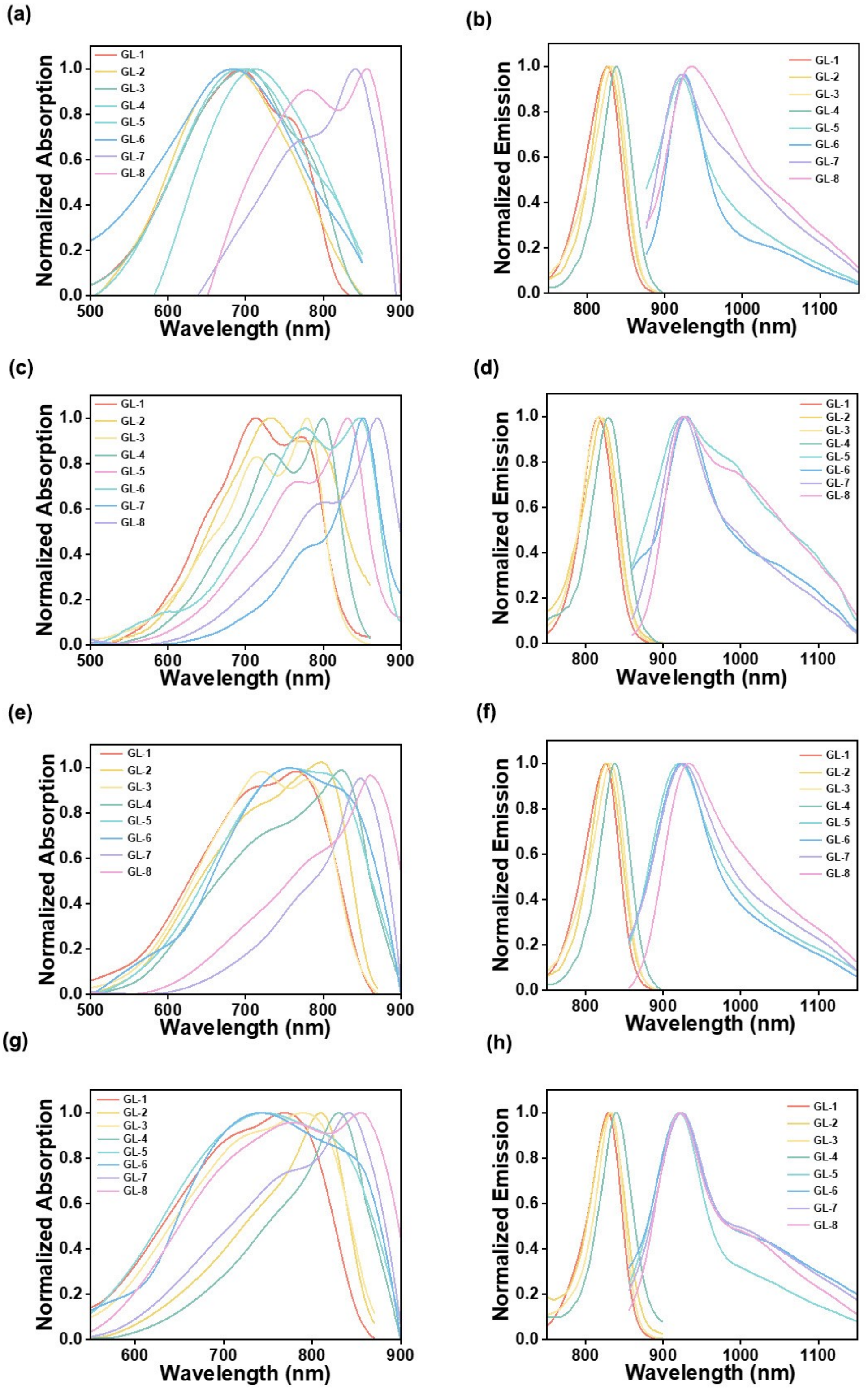

Figure S1. Normalized absorption and fluorescence emission spectra of **GL-1–8** dyes in MeCN (a and b), CH_2Cl_2 (c and d), MeOH (e and f), MeCN/PBS (1:1 = v/v, pH = 7.4) (g and h), respectively.

Table S1. Photophysical properties of GL-1–8 in MeCN

Compound	λ_{max} (nm)	ε_{max} (10^4 M^{-1} cm^{-1})	λ_{em} (nm)	Φ (%)	Stokes shift (nm)
GL-1	682	4.95	810	0.25	128
GL-2	689	4.10	822	0.20	121
GL-3	687	2.50	820	0.14	133
GL-4	710	1.89	832	0.04	122
GL-5	687	7.60	918	0.05	231
GL-6	680	3.20	922	0.04	242
GL-7	844	2.80	927	0.08	83
GL-8	863	2.90	937	0.09	74

Table S2. Photophysical properties of GL-1–8 in CH_2Cl_2

Compound	λ_{max} (nm)	ε_{max} (10^4 M^{-1} cm^{-1})	λ_{em} (nm)	Φ (%)	Stokes shift (nm)
GL-1	718	4.37	816	0.30	83
GL-2	733	4.28	818	0.33	46
GL-3	778	2.75	821	0.45	43
GL-4	800	3.21	830	0.07	34
GL-5	833	7.40	925	0.31	92
GL-6	835	2.80	925	0.13	90
GL-7	852	4.50	930	0.21	78
GL-8	870	4.40	927	0.31	57

Table S3. Photophysical properties of GL-1–8 in MeOH

Compound	λ_{max} (nm)	ε_{max} (10^4 M^{-1} cm^{-1})	λ_{em} (nm)	Φ (%)	Stokes shift (nm)
GL-1	766	3.20	825	0.31	107
GL-2	799	3.34	827	0.11	63
GL-3	723/780	3.02	832	0.21	36/109
GL-4	824	3.02	839	0.08	15
GL-5	753/807	2.40	920	0.20	167/113
GL-6	764/818	1.80	923	0.02	159/105
GL-7	849	3.80	927	0.33	78
GL-8	860	3.50	936	0.34	76

Table S4. Photophysical properties of GL-1–8 in MeCN/PBS (1:1 = v/v, pH = 7.4)

Compound	λ_{max} (nm)	ε_{max} (10^4 M^{-1} cm^{-1})	λ_{em} (nm)	Φ (%)	Stokes shift (nm)
GL-1	772	5.40	826	0.24	60
GL-2	810	4.80	831	0.22	40
GL-3	790	2.40	835	0.20	27
GL-4	834	2.21	840	0.41	11
GL-5	761/820	1.60	919	0.09	184/99
GL-6	842	1.20	920	0.11	78
GL-7	845	3.10	923	0.08	78
GL-8	860	1.90	921	0.09	61

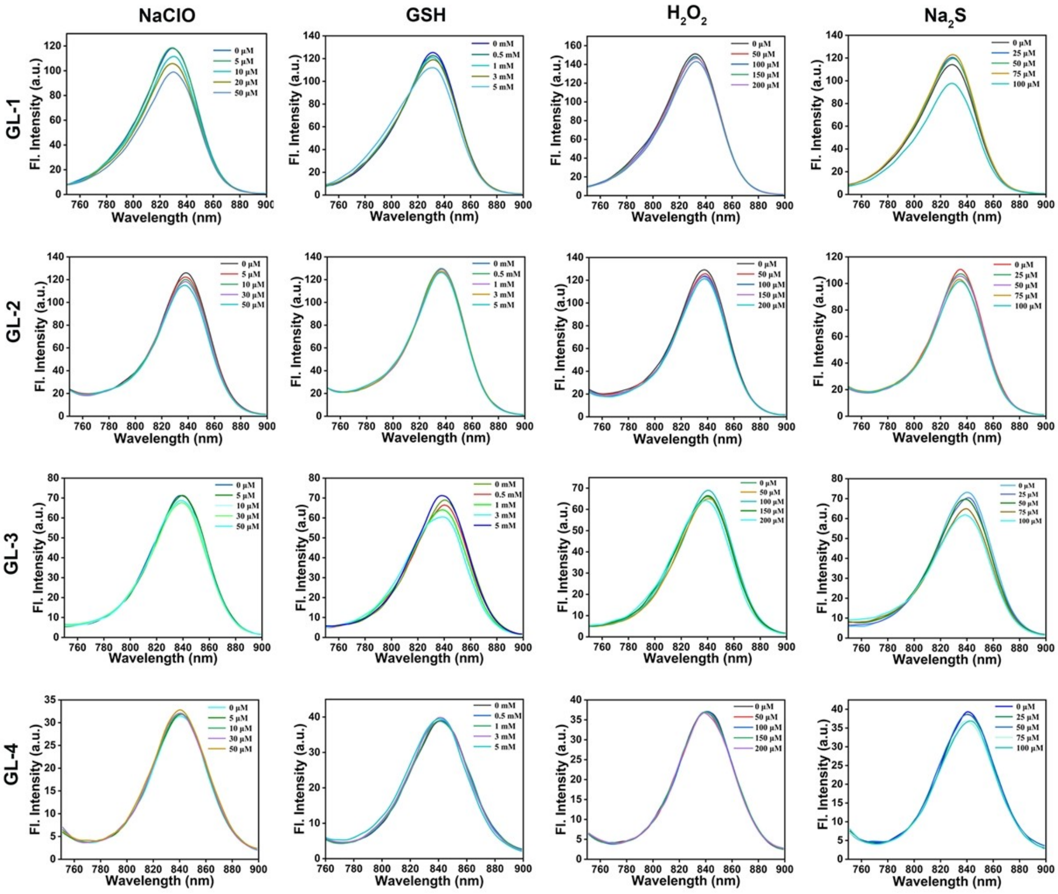

Figure S2. The fluorescence spectra of compounds **GL-1–4** (10 µM) were analyzed after treatment with various agents in MeCN/PBS (1:1 = v/v, pH = 7.4). The concentrations of the agents were as follows: NaClO (0 – 50 µM), GSH (0 – 5 mM), H_2O_2 (0 – 200 µM), and Na_2S (0 – 100 µM). Data were recorded 15 min after the addition of the analytes.

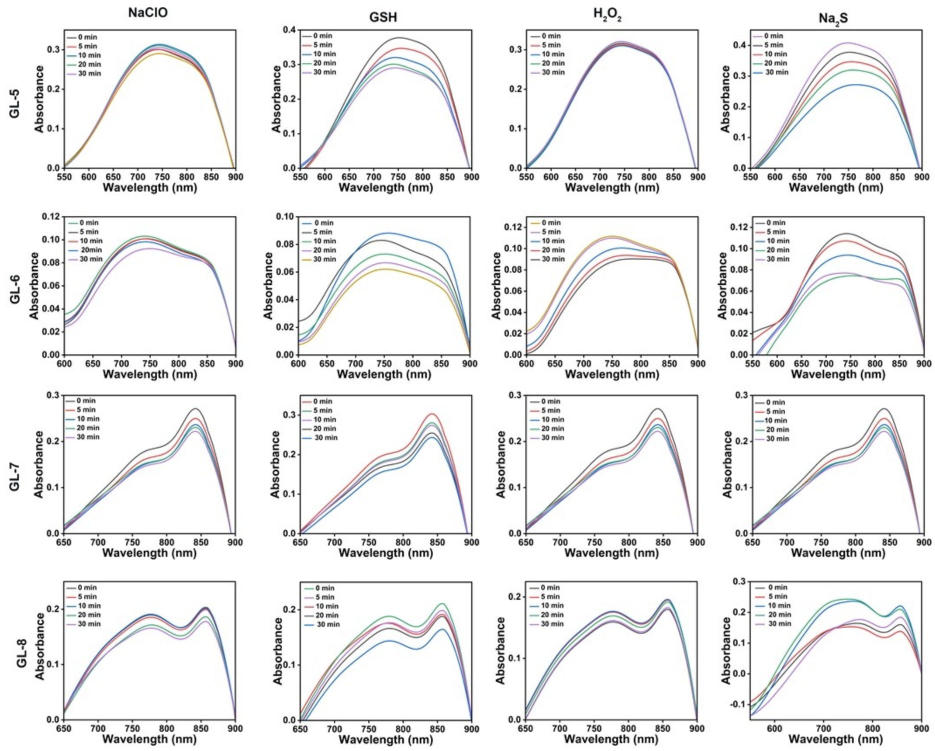

Figure S3. The time-dependent UV absorption spectra of **GL-5–8** (10 μM) were obtained in MeCN/PBS (1:1 = v/v, pH = 7.4), in the presence of various agents: 25 μM NaClO, 1 mM GSH, 100 μM H_2O_2, and 50 μM Na_2S.

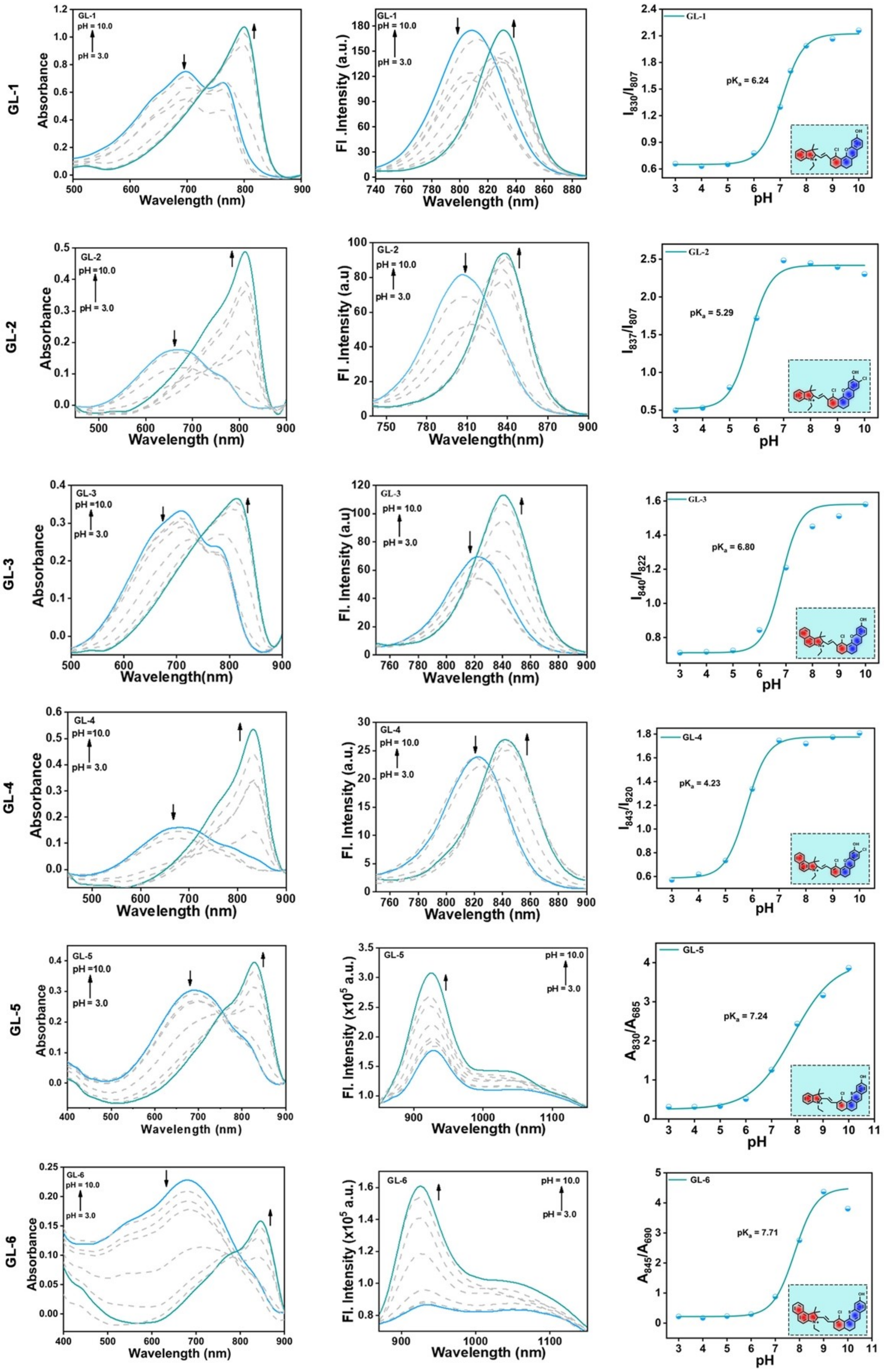

Figure S4. The UV absorption and fluorescence spectra of **GL-1–6** (10 μM) were measured in MeCN/PBS (1:1 = v/v) at pH values ranging from 3.0 to 10.0.

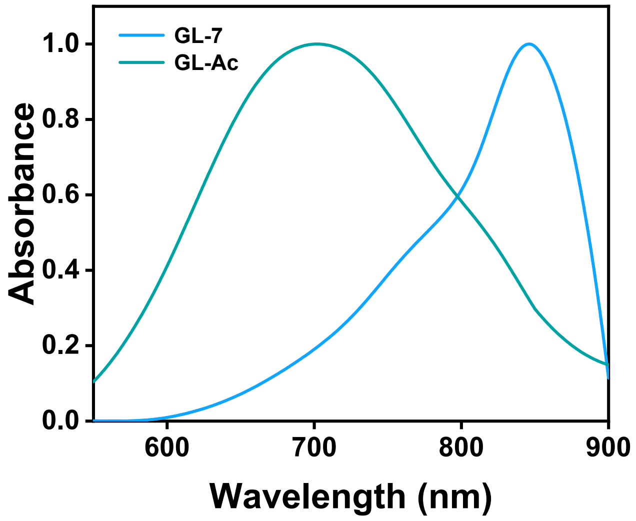

Figure S5. Normalized absorption spectra of **GL-7** and **GL-Ac** in MeOH.

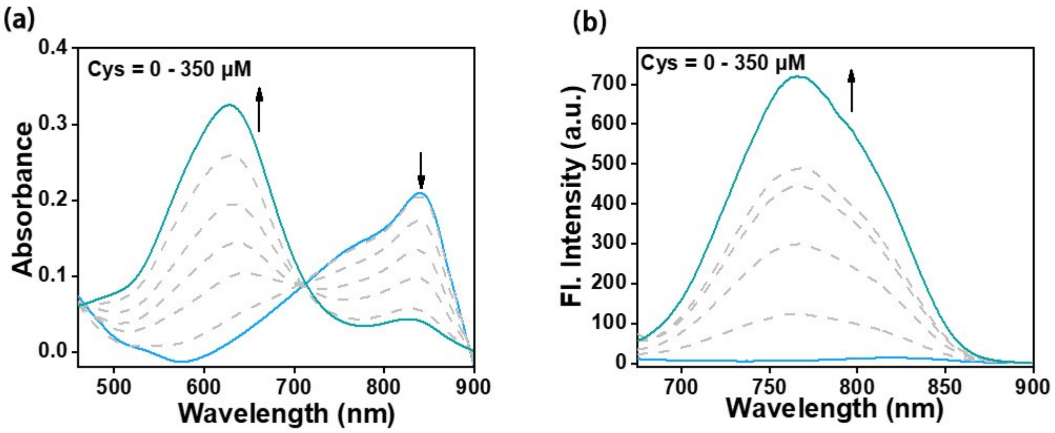

Figure S6. (a) UV absorption spectrometric titration experiments of **GL-OH** with different concentrations of Cys. (b) Fluorescence emission spectra of **GL-OH** with different concentrations of Cys (λ_{ex} = 640 nm).

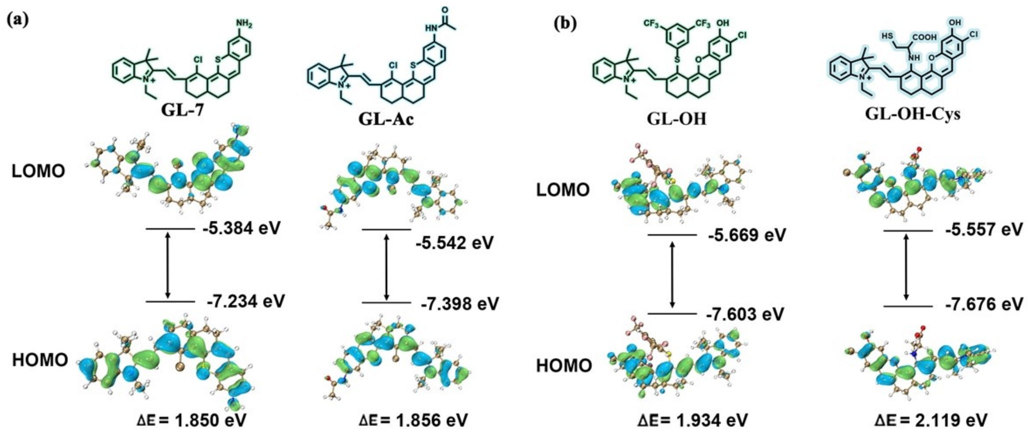

Figure S7. (a) Density functional theory (DFT) calculations of **GL-7** and **GL-Ac** optimized at the B3LYP/6-31 + G(d) level of theory. (b) DFT calculations of **GL-OH** and **GL-OH-Cys** optimized at the B3LYP/6-31 + G(d) level of theory.

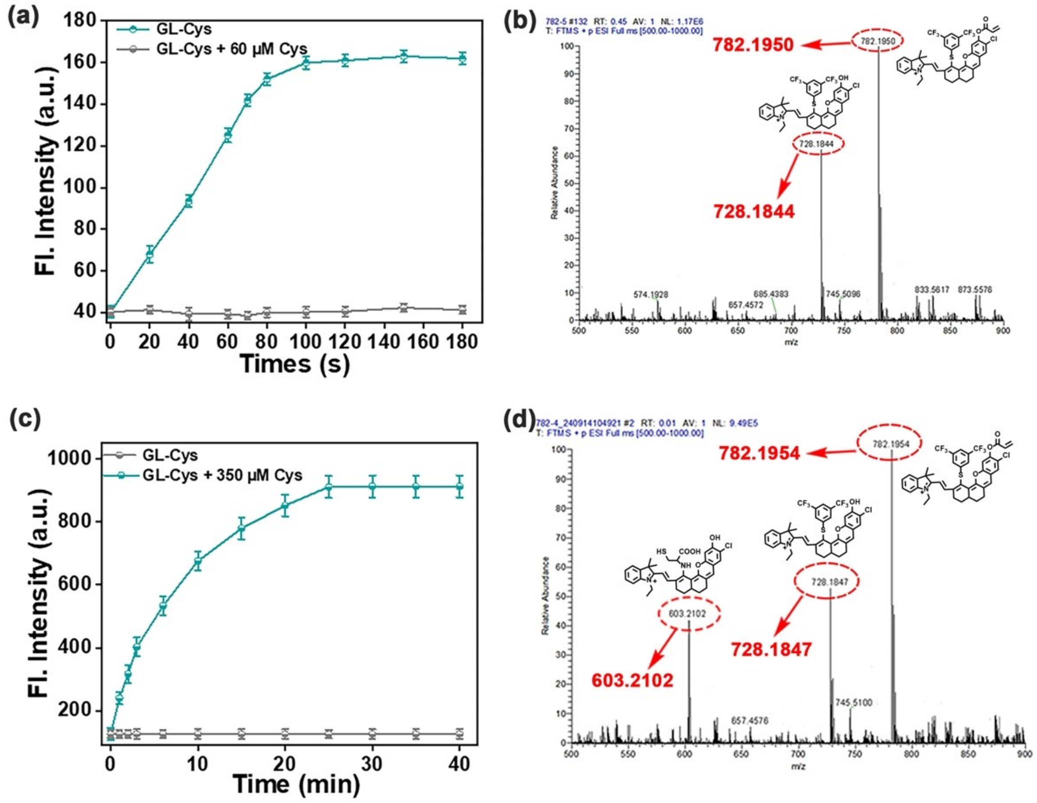

Figure S8. (a) Time-dependent fluorescence responses at 830 nm of **GL- Cys** (10 μM) to low concentrations Cys (60 μM). (b) High-resolution mass spectrometry (HRMS) of the reaction solution of **GL-Cys** (10 μM) with Cys (60 μM). (c) Time-dependent fluorescence responses at 765 nm of **GL-Cys** (10 μM) to high concentrations Cys (350 μM). (d) HRMS of the reaction solution of **GL-Cys** (10 μM) with Cys (350 μM).

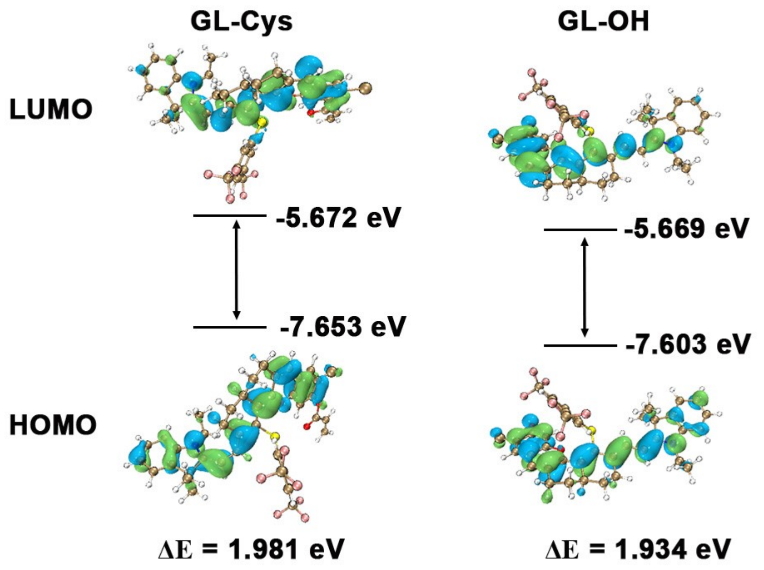

Figure S9. Based on DFT calculations performed at the B3LYP/6-31 + G(d) level, we have theoretically calculated the HOMO and the LUMO of the **GL-Cys** and **GL-OH** molecule.

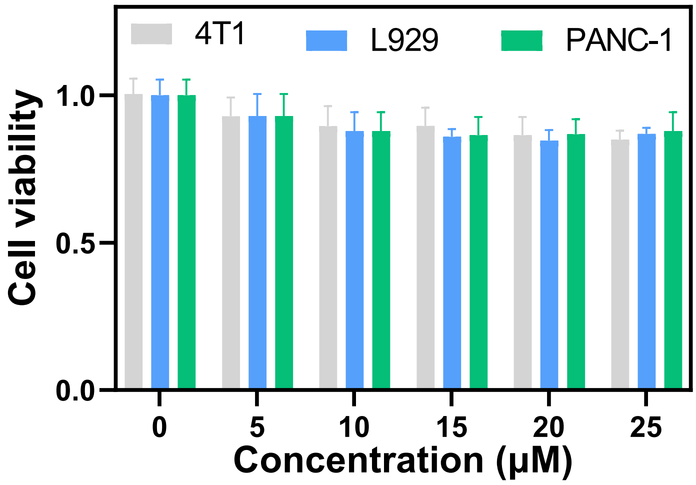

Figure S10. The cytotoxicity of the probe **GL-Cys** was assessed on different cell lines, including 4T1 cells, L929 cells and PANC-1 cells, using the MTT assay. The results are expressed as the mean ± standard deviation of six independent measurements.

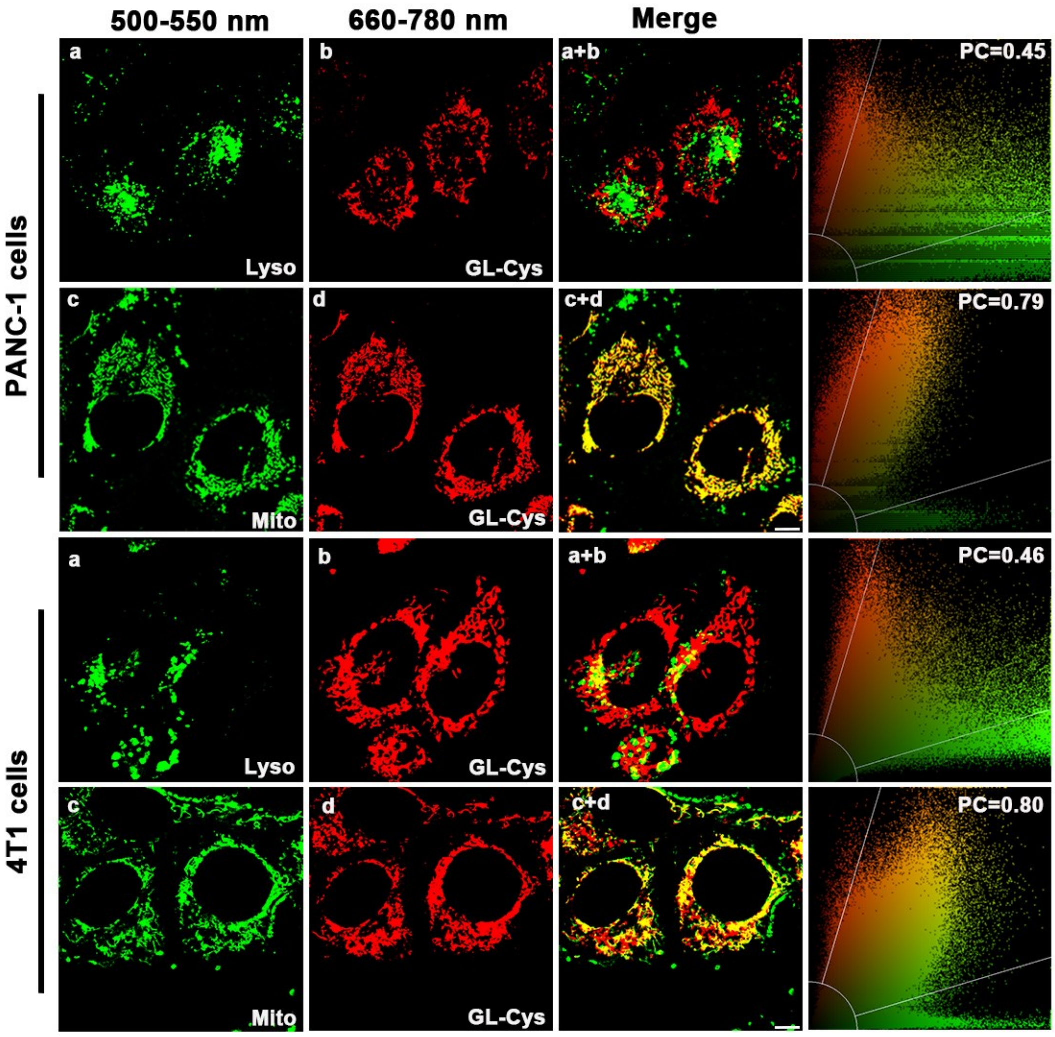

Figure S11. Confocal microscopic images demonstrated the colocalization of **GL-Cys** with various commercially available organelle trackers in PANC-1 and 4T1 cells. The cells were incubated with **GL-Cys** (10 μM) for 15 min, followed by treatment with Mito Tracker Green (500 nM) and Lysotracker Green (500 nM) for an additional 15 min. The red channel: 660 – 780 nm, λ_{ex} = 638 nm, for **GL-Cys**; Green channel: 500 – 550 nm, λ_{ex} = 488 nm, for Mito-tracker or Lysotracker. Scale bar = 20 μm.

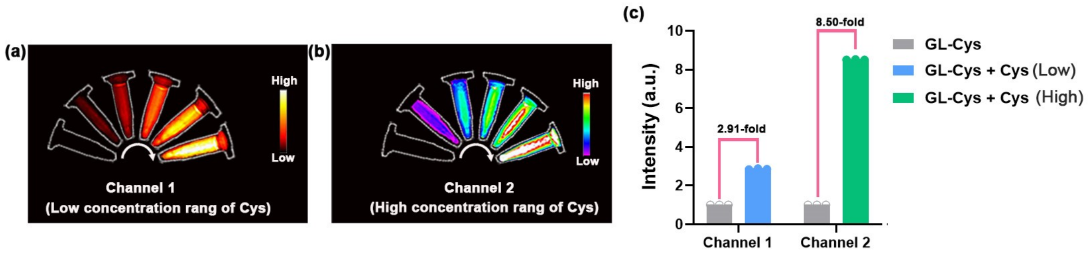

Figure S12. *In vitro* dual-channel fluorescence imaging was conducted. (a) The **GL-Cys** (0.5 mM) to low concentrations of Cys (λ_{ex} = 720 nm with filter 790 nm). (b) The **GL-Cys** (0.5 mM) to high concentrations of Cys (λ_{ex} = 690 nm with filter 750 nm). (c) Relative fluorescence signal of **GL-Cys** upon the addition of Cys (n = 3).

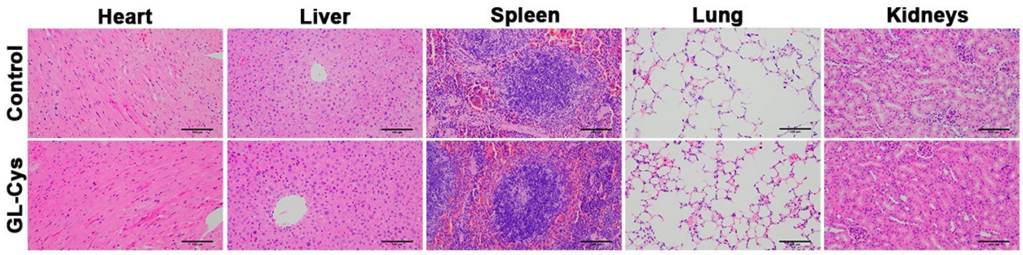

Figure S13. Histological analyses of various organs from healthy mice following tail intravenous injection of saline (100 μL) (control group), **GL-Cys** solution (100 μM, 100 μL) (experimental group), over a 24 h period. Scale bar: 100 μm.

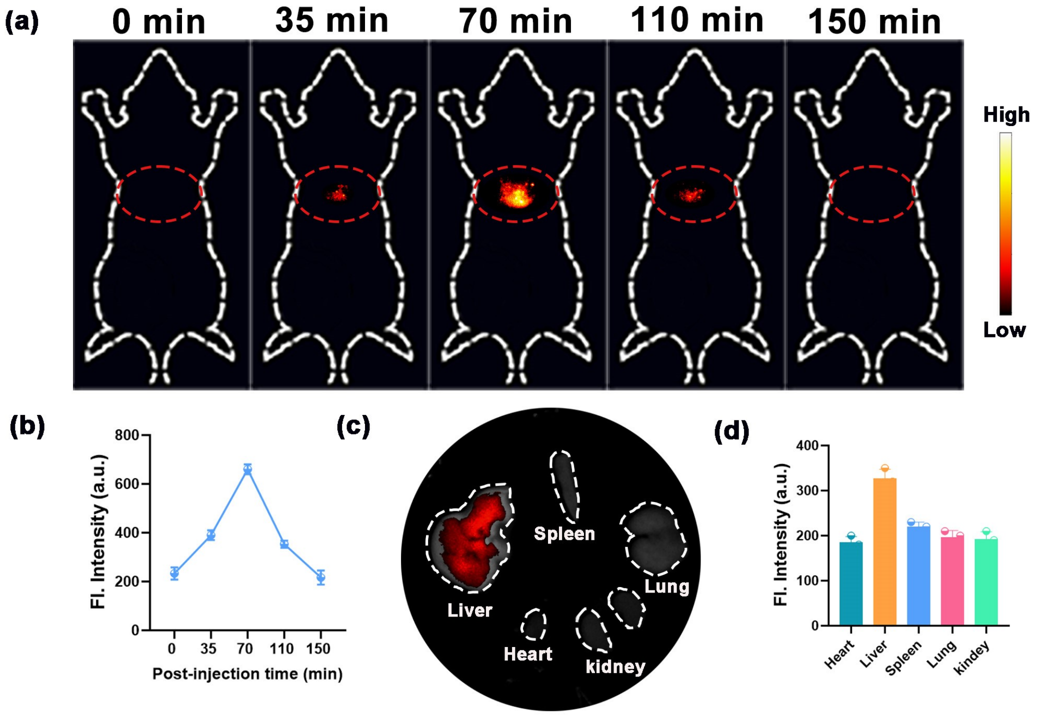

Figure S14. Fluorescence imaging of the mice at different time points after injection of **GL-Cys**. (b) Fluorescence intensity in image (a). (c) Fluorescent images for the organs (which are from various groups at 150 min upon tail i.v. injection of the **GL-Cys**, including liver, kidney, spleen, heart, and lung). (d) Fluorescence intensity in image (c). Fluorescence images were obtained under λex = 690 nm, with a filter at λ = 750 nm. All data were presented as mean ± SD (n = 3).

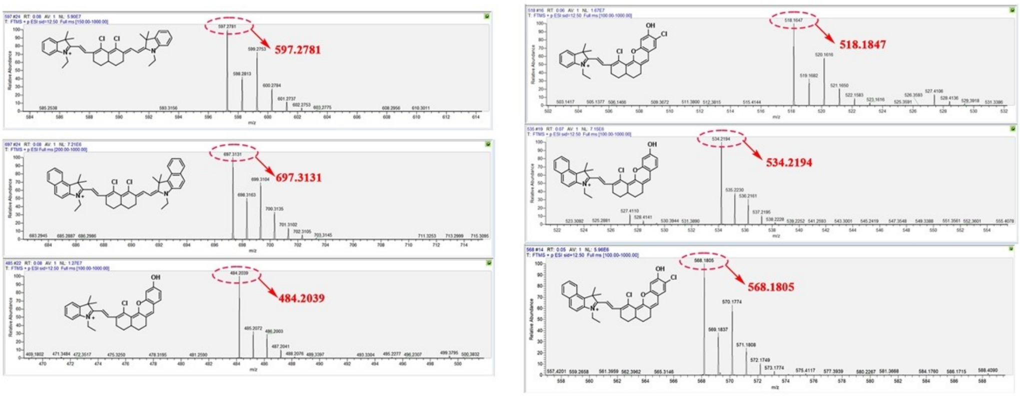

Figure S15. HRMS of compounds **Cy-1–2** and **GL-1–4.**

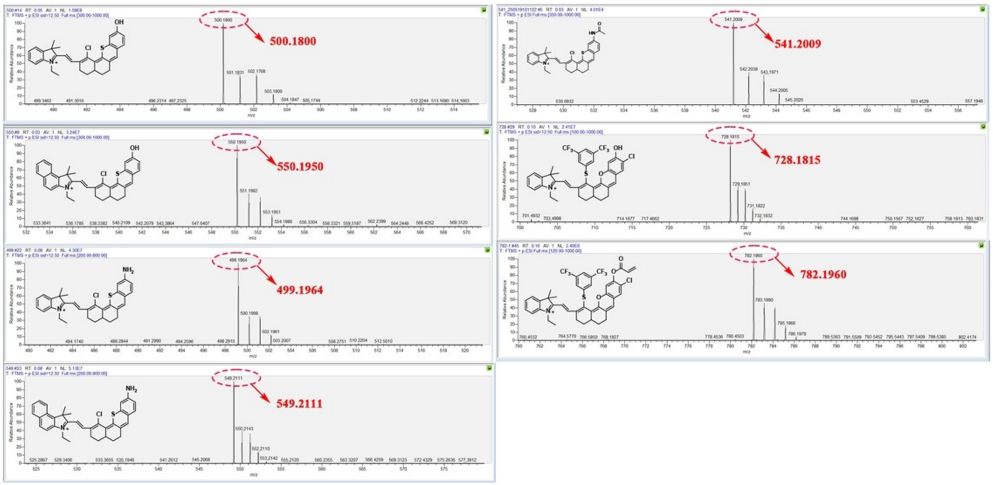

Figure S16. HRMS of compounds **GL-5–8**, **GL-Ac**, **GL-OH** and **GL-Cys**.

17. NMR spectra

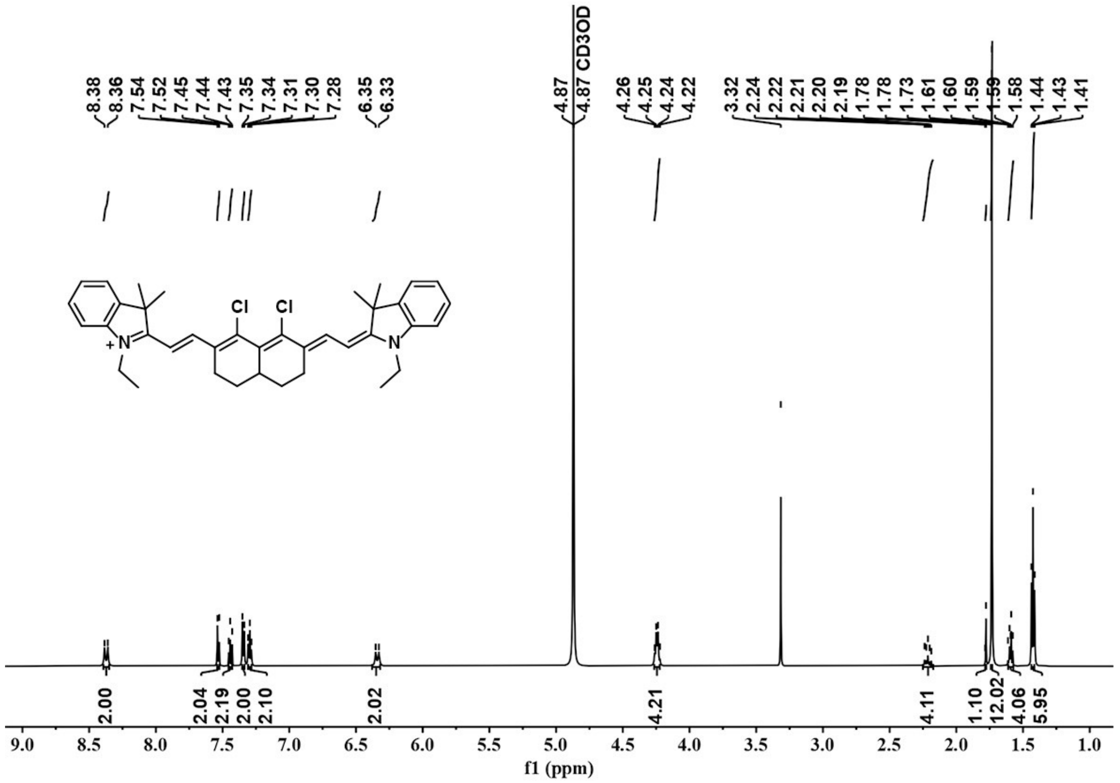

Figure S17. ^{1}H NMR spectrum of **Cy-1** (Methanol-d_4).

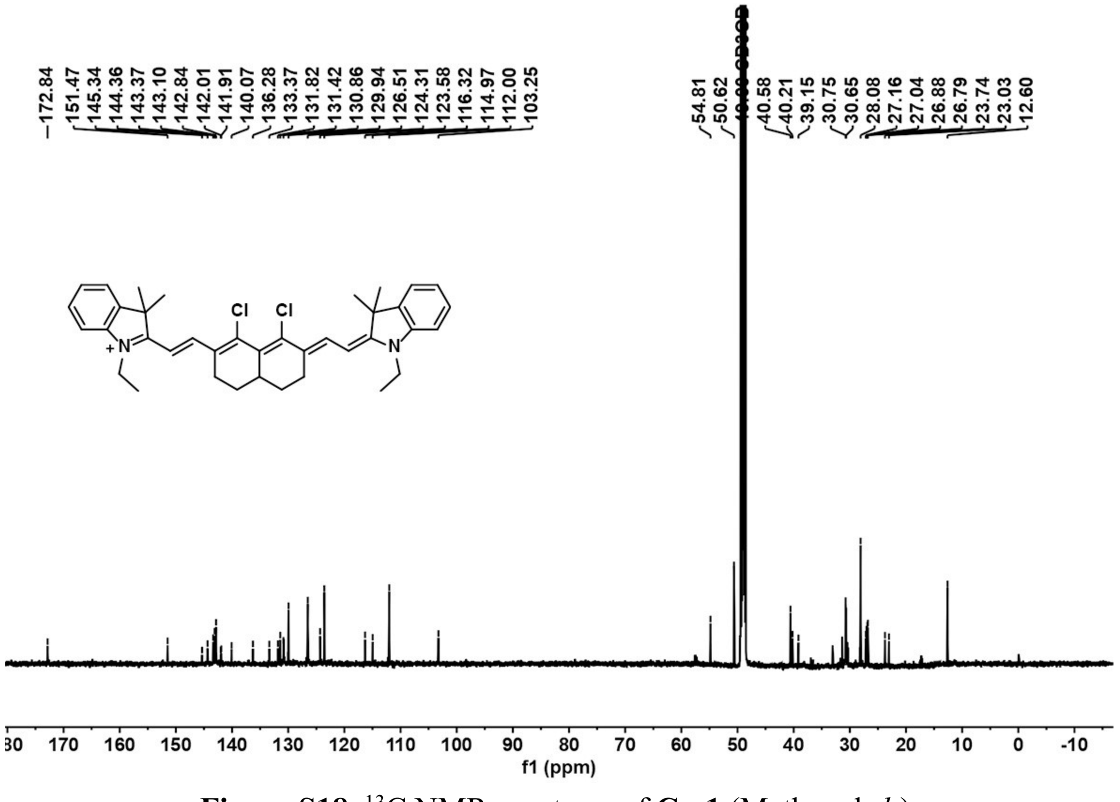

Figure S18. ^{13}C NMR spectrum of **Cy-1** (Methanol-d_4).

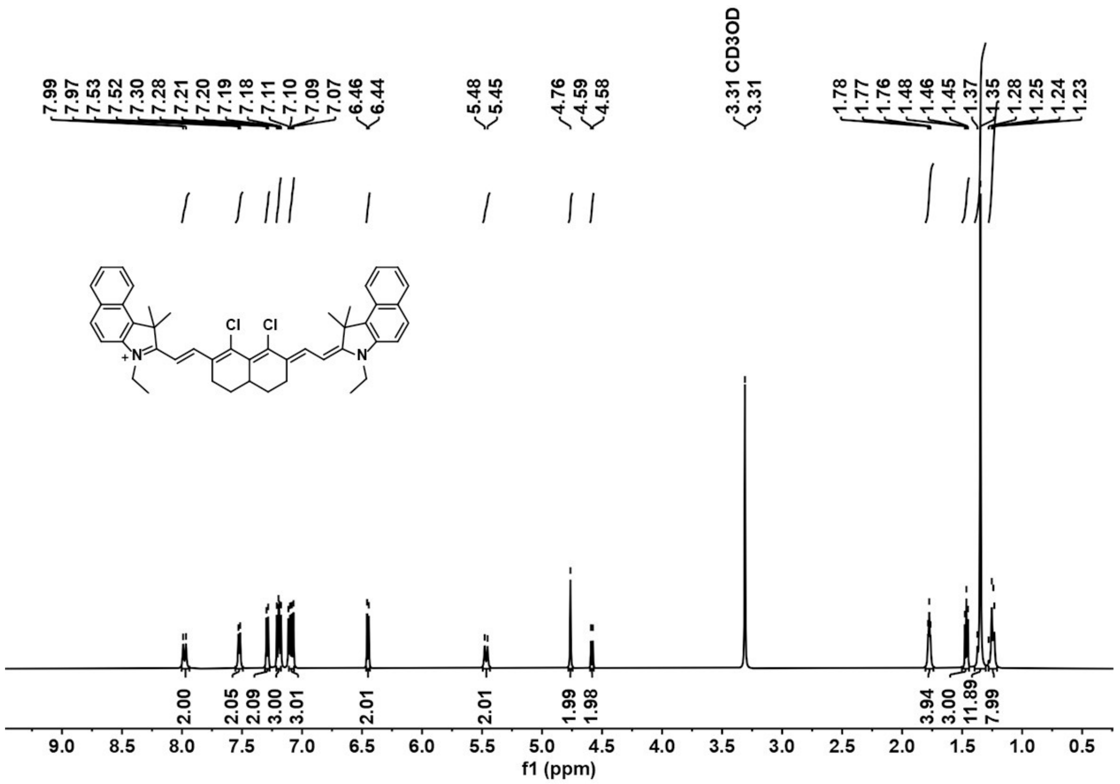

Figure S19. ^{1}H NMR spectrum of **Cy-2** (Methanol-d_4).

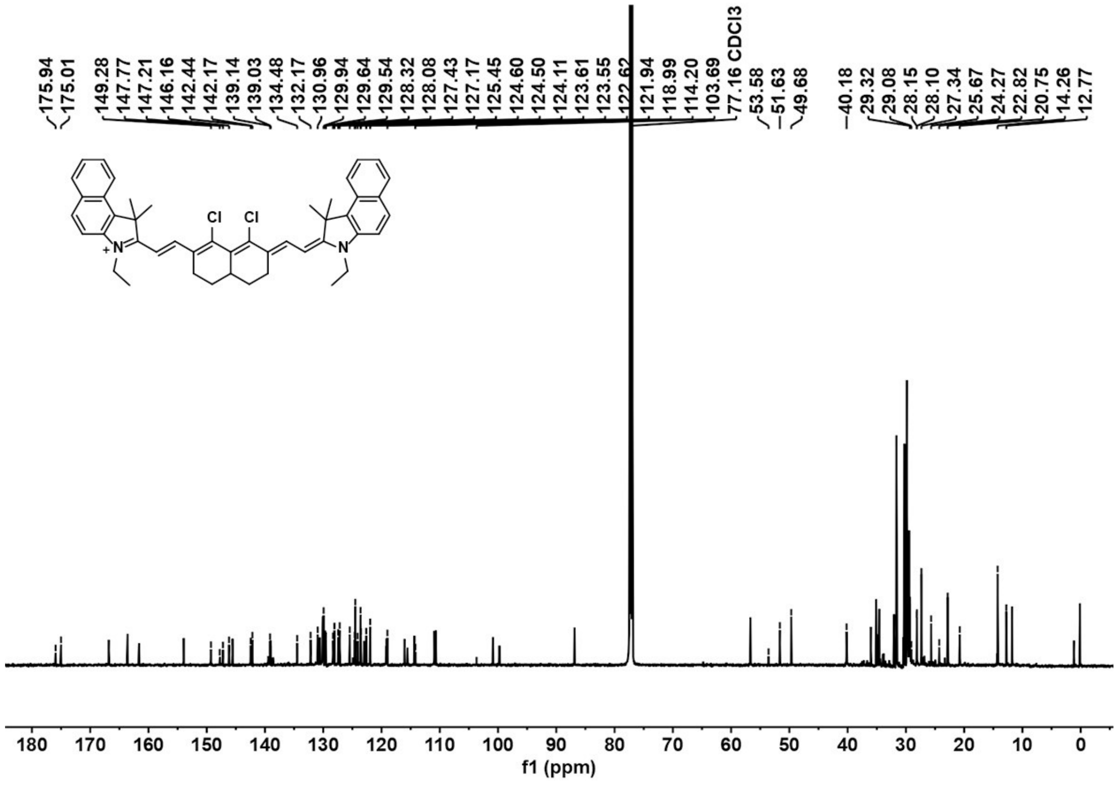

Figure S20. ^{13}C NMR spectrum of **Cy-2** ($CDCl_3$).

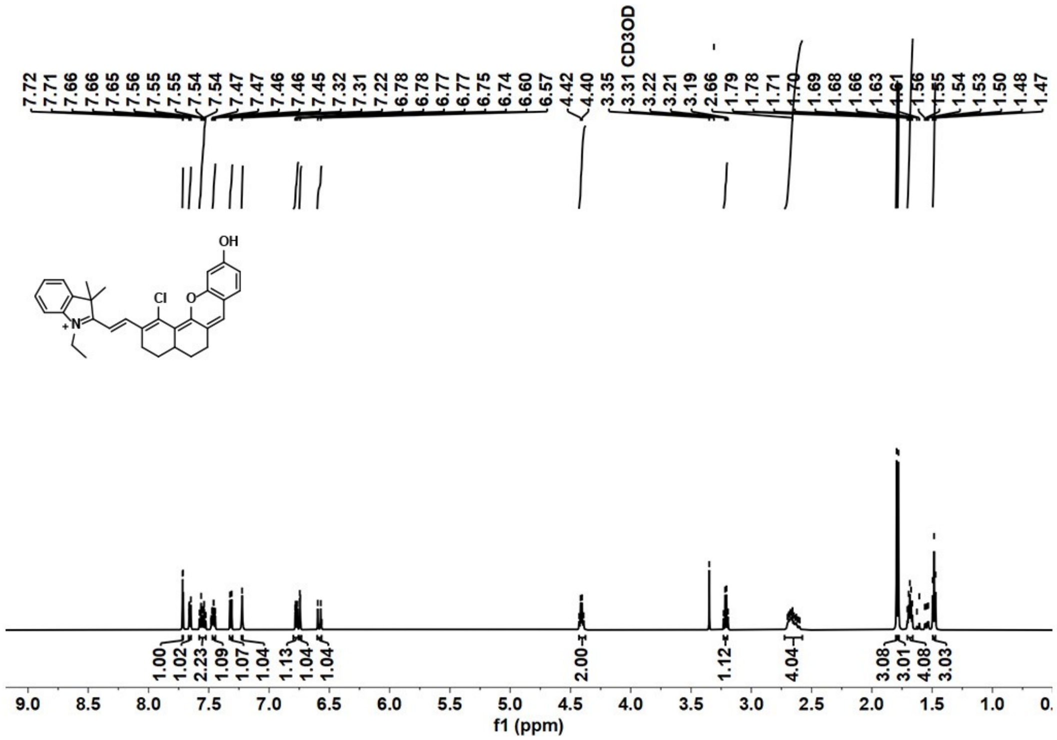

Figure S21. ^{1}H NMR spectrum of **GL-1** (Methanol-d_4).

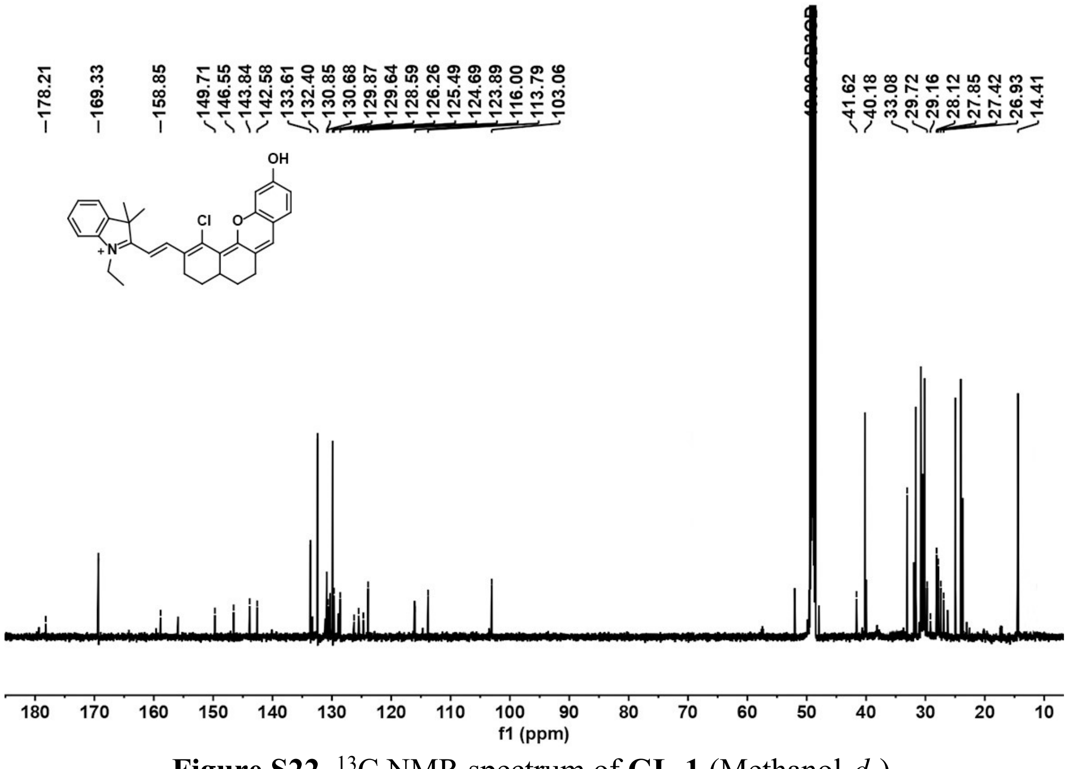

Figure S22. ^{13}C NMR spectrum of **GL-1** (Methanol-d_4).

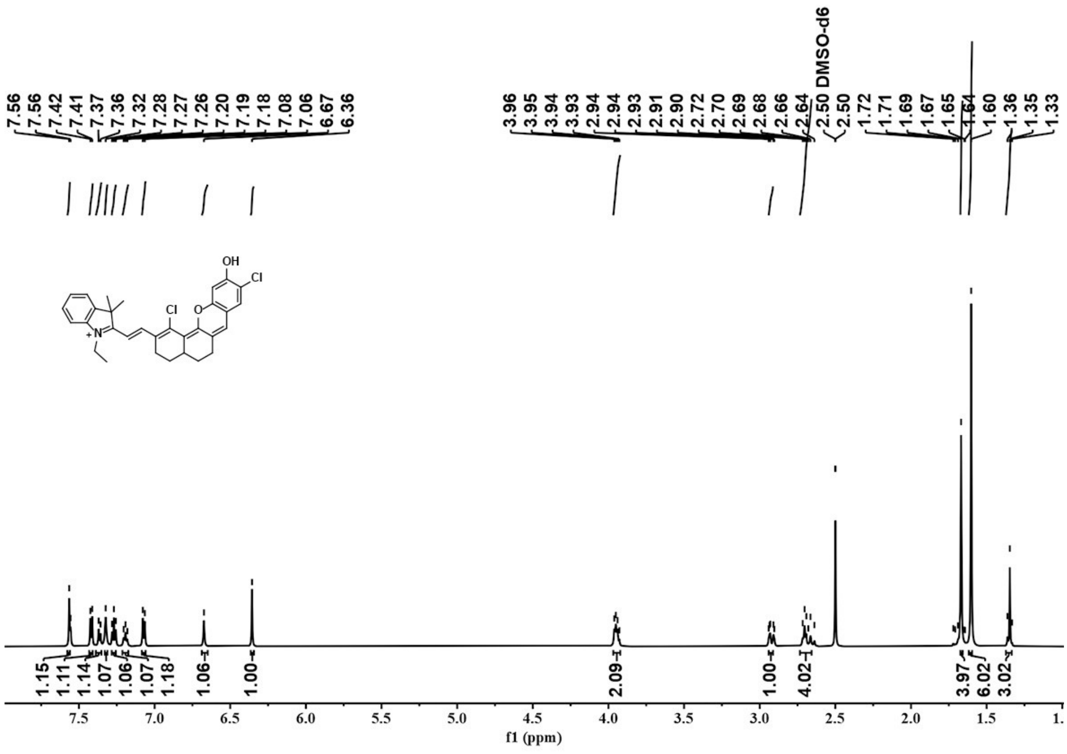

Figure S23. ^{1}H NMR spectrum of **GL-2** (DMSO-d_6).

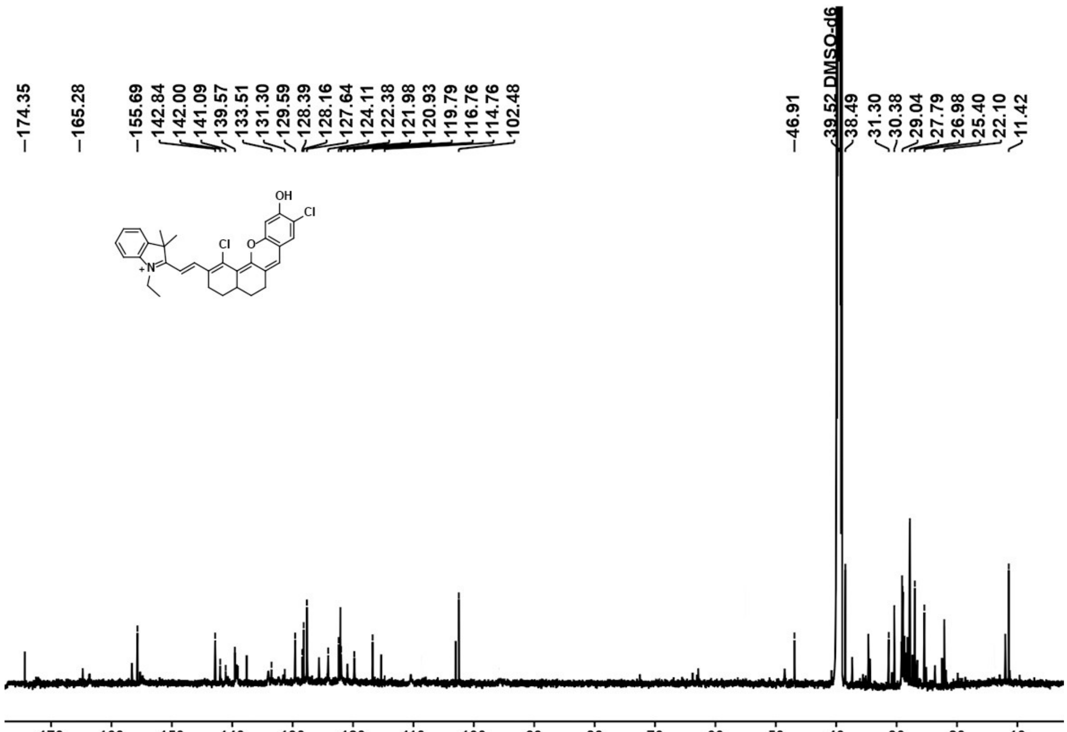

Figure S24. ^{13}C NMR spectrum of **GL-2** (DMSO-d_6).

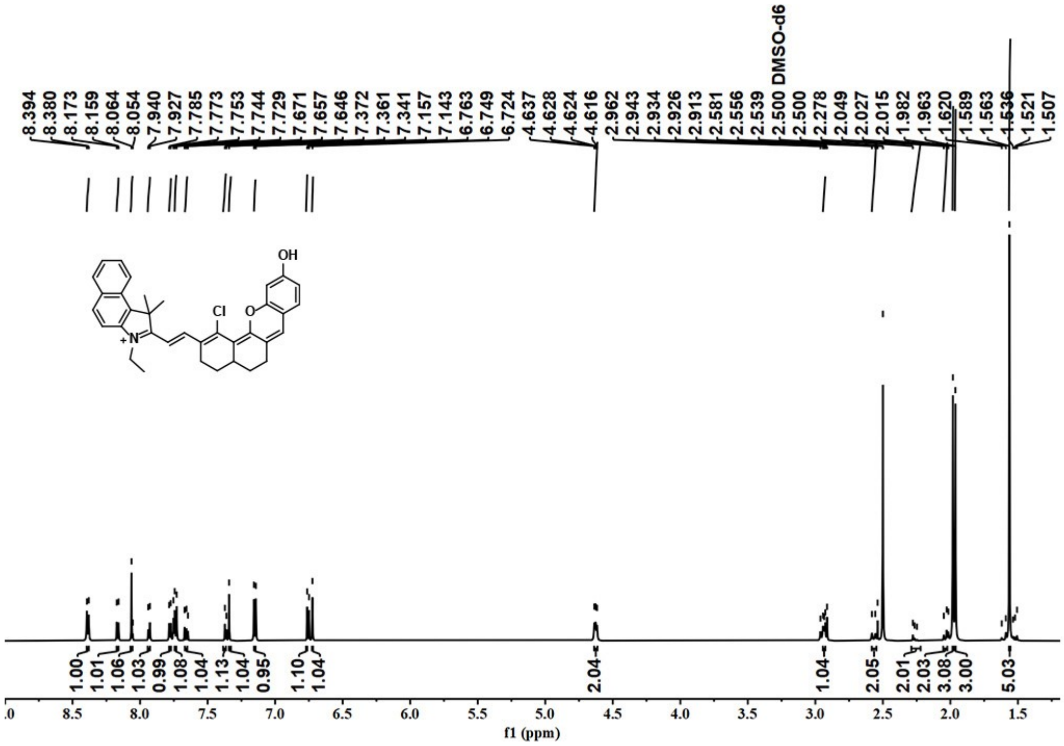

Figure S25. ^{1}H NMR spectrum of **GL-3** (DMSO-d_6).

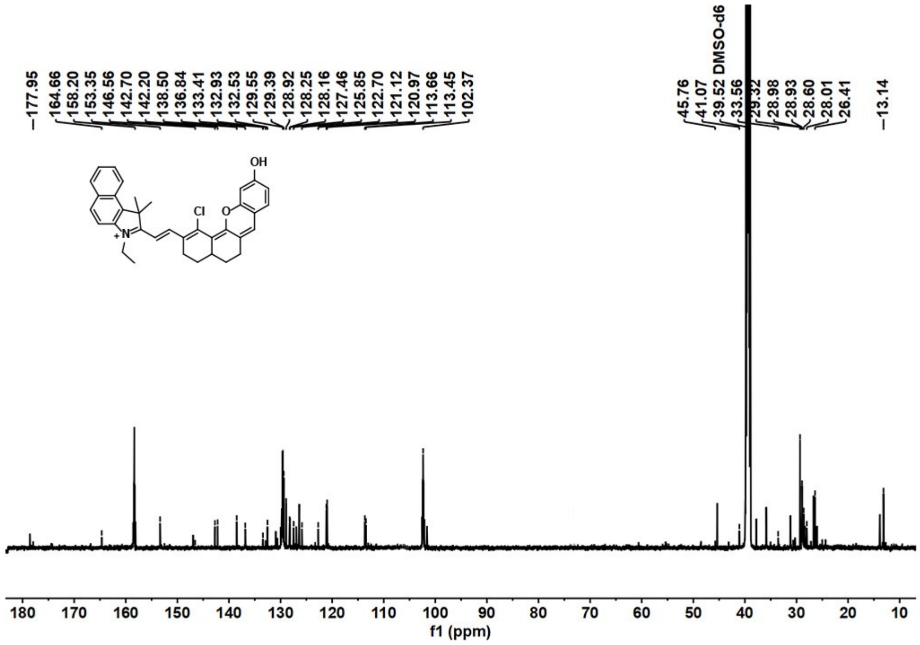

Figure S26. ^{13}C NMR spectrum of **GL-3** (DMSO-d_6).

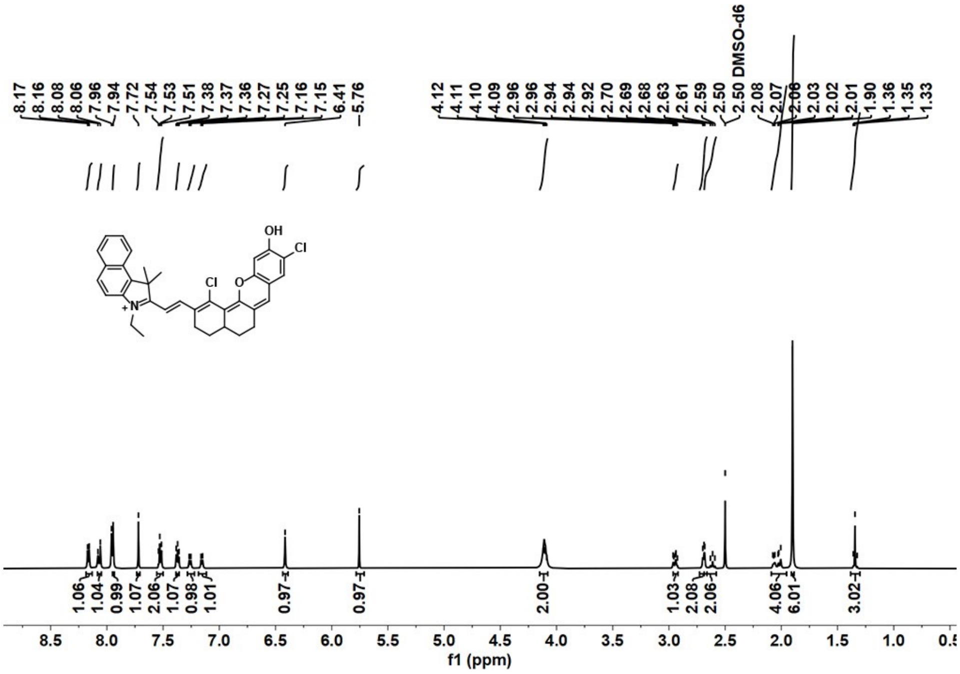

Figure S27. ^{1}H NMR spectrum of **GL-4** (DMSO-d_6).

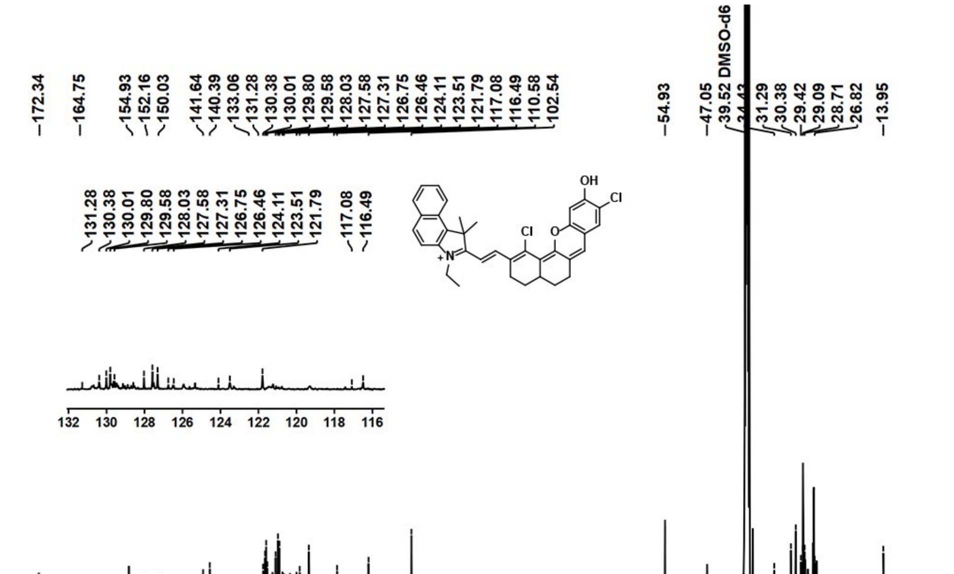

Figure S28. ^{13}C NMR spectrum of **GL-4** (DMSO-d_6).

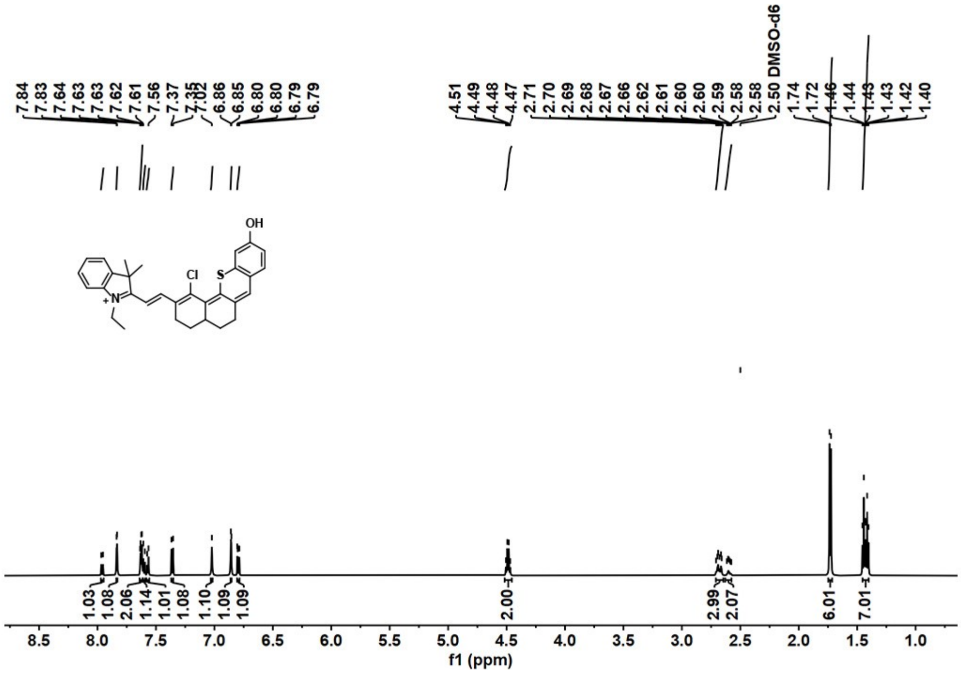

Figure S29. ^{1}H NMR spectrum of **GL-5** (DMSO-d_6).

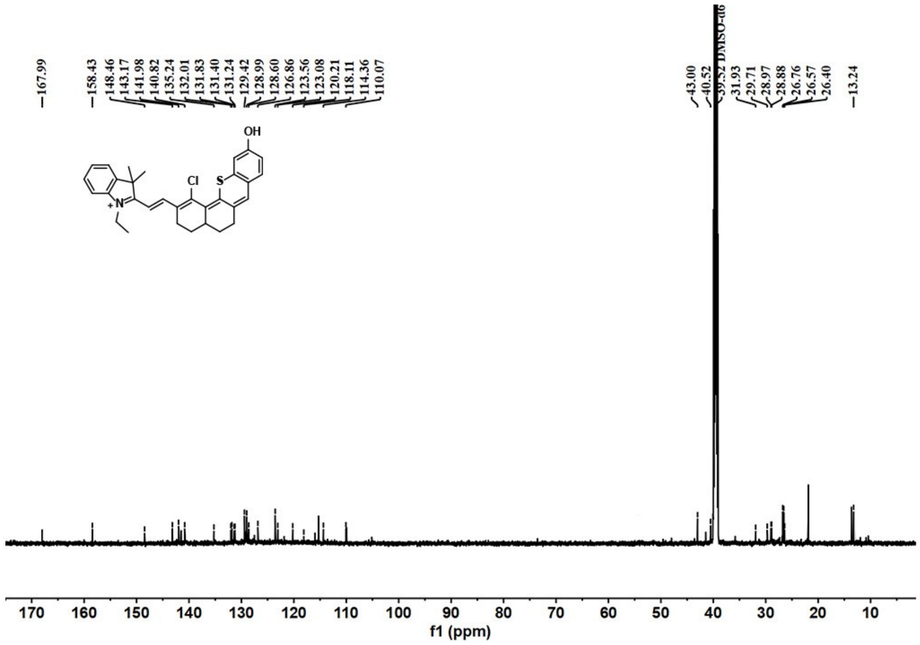

Figure S30. ^{13}C NMR spectrum of **GL-5** (DMSO-d_6).

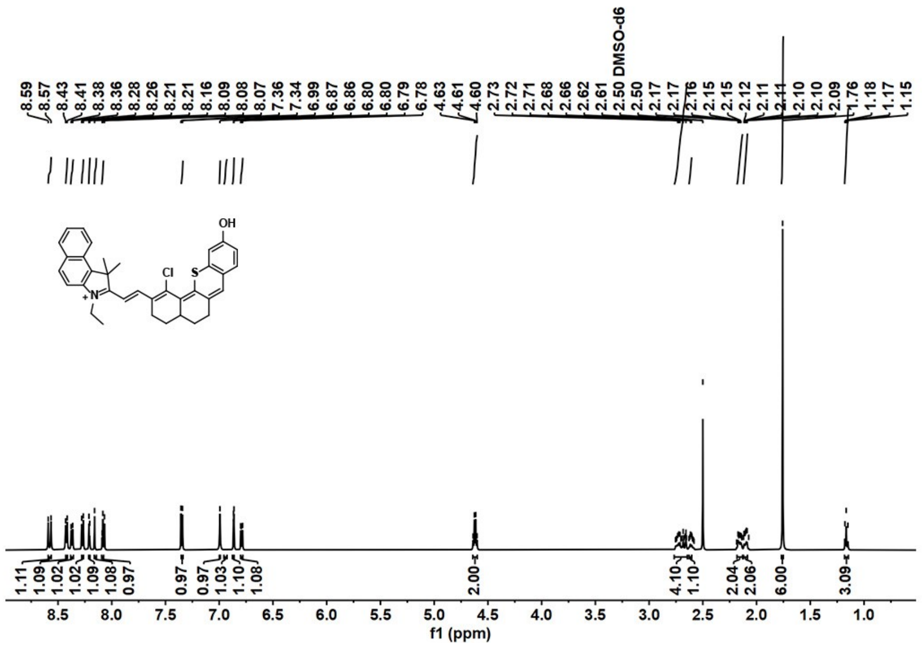

Figure S31. ^{1}H NMR spectrum of **GL-6** (DMSO-d_6).

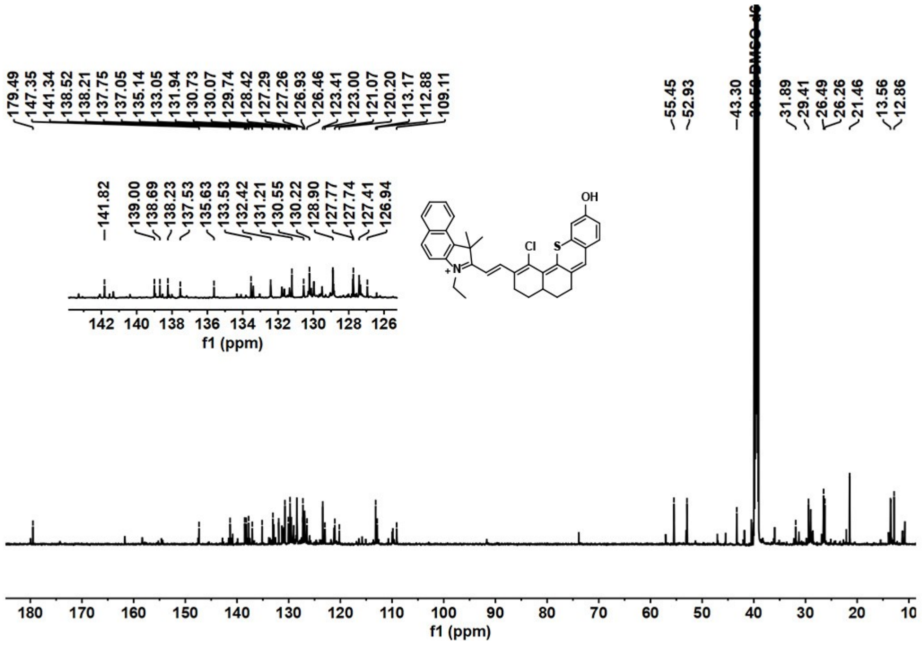

Figure S32. ^{13}C NMR spectrum of **GL-6** (DMSO-d_6).

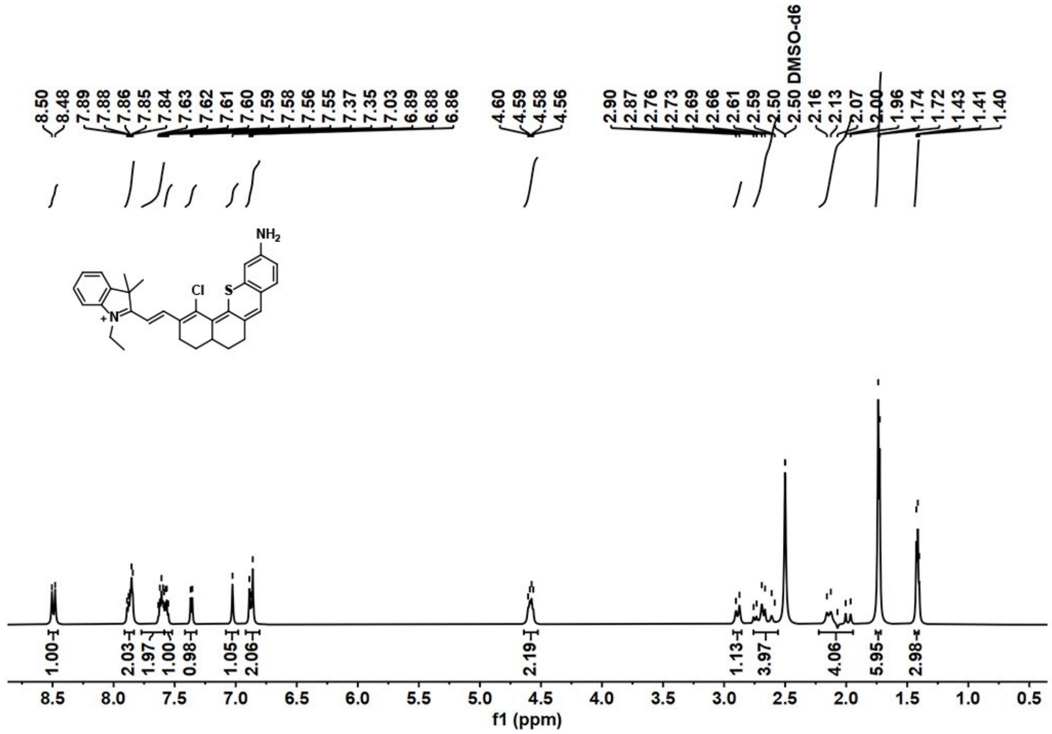

Figure S33. ^{1}H NMR spectrum of **GL-7** (DMSO-d_6).

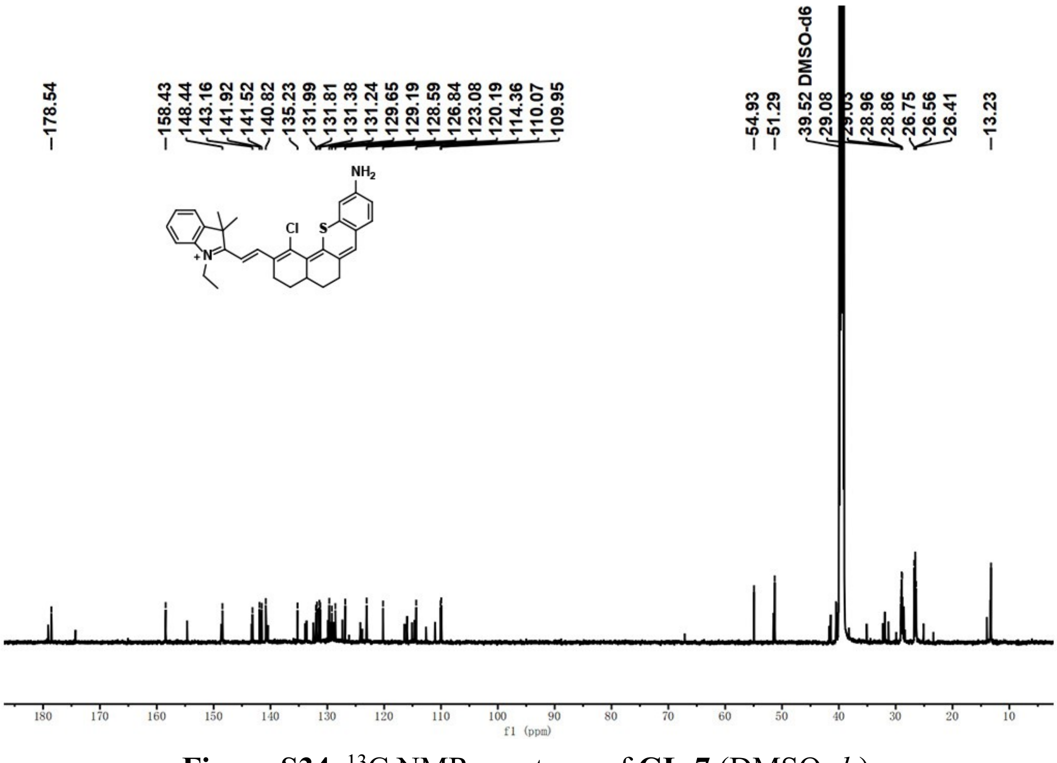

Figure S34. ^{13}C NMR spectrum of **GL-7** (DMSO-d_6).

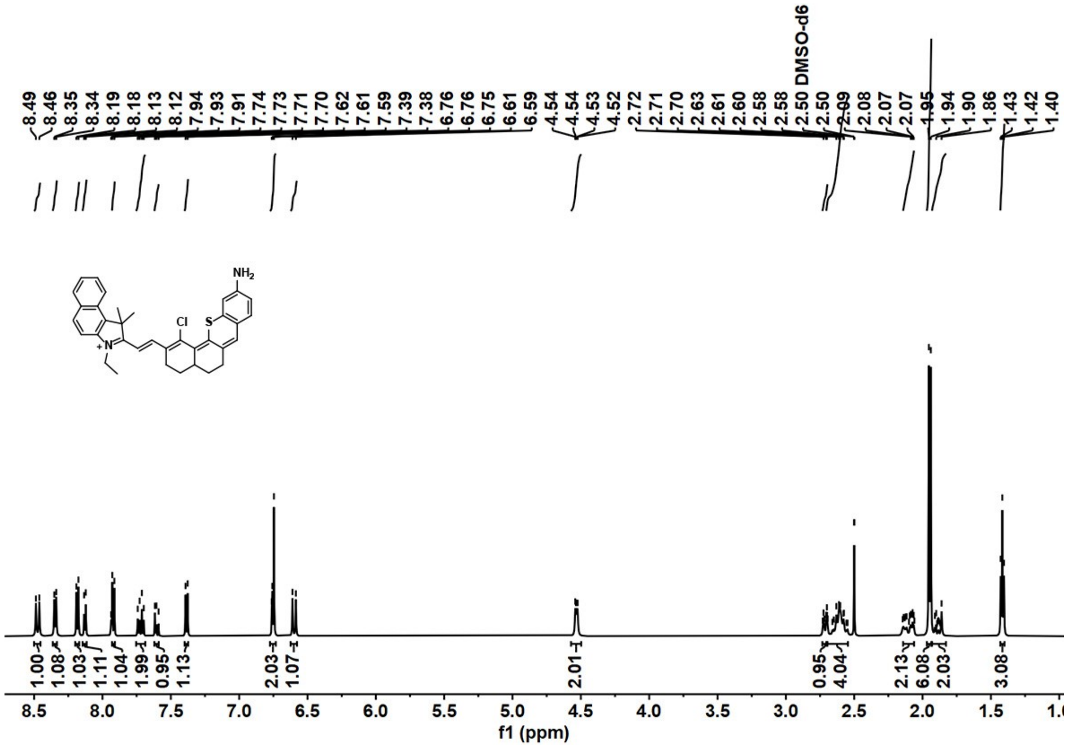

Figure S35. ^{1}H NMR spectrum of **GL-8** (DMSO-d_6).

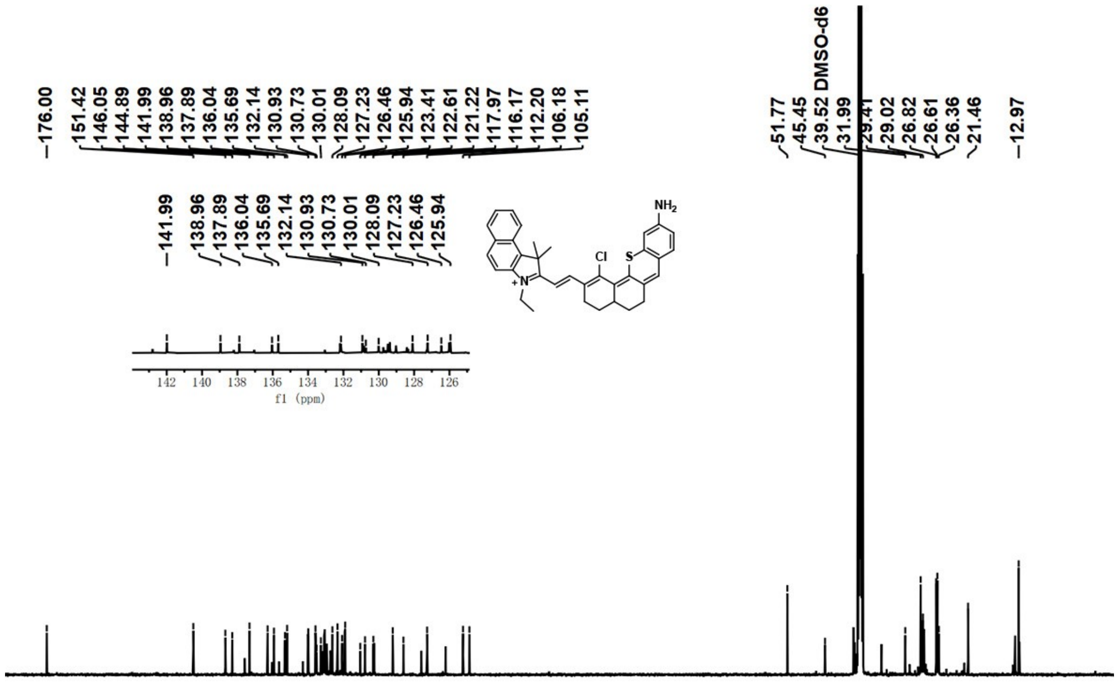

Figure S36. ^{13}C NMR spectrum of **GL-8** (DMSO-d_6).

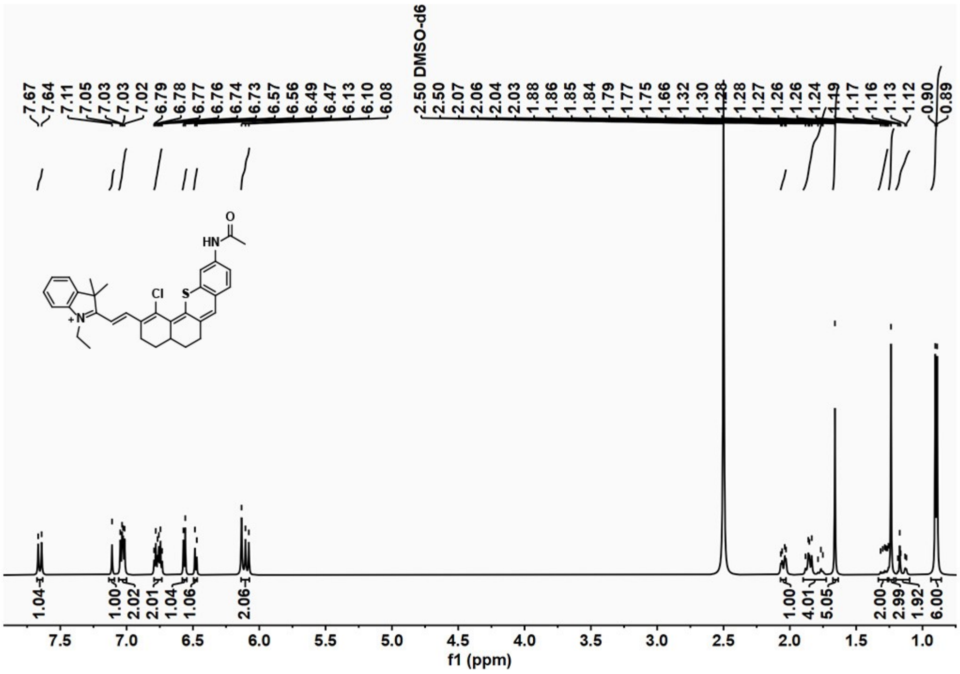

Figure S37. ^{1}H NMR spectrum of **GL-Ac** (DMSO-d_6).

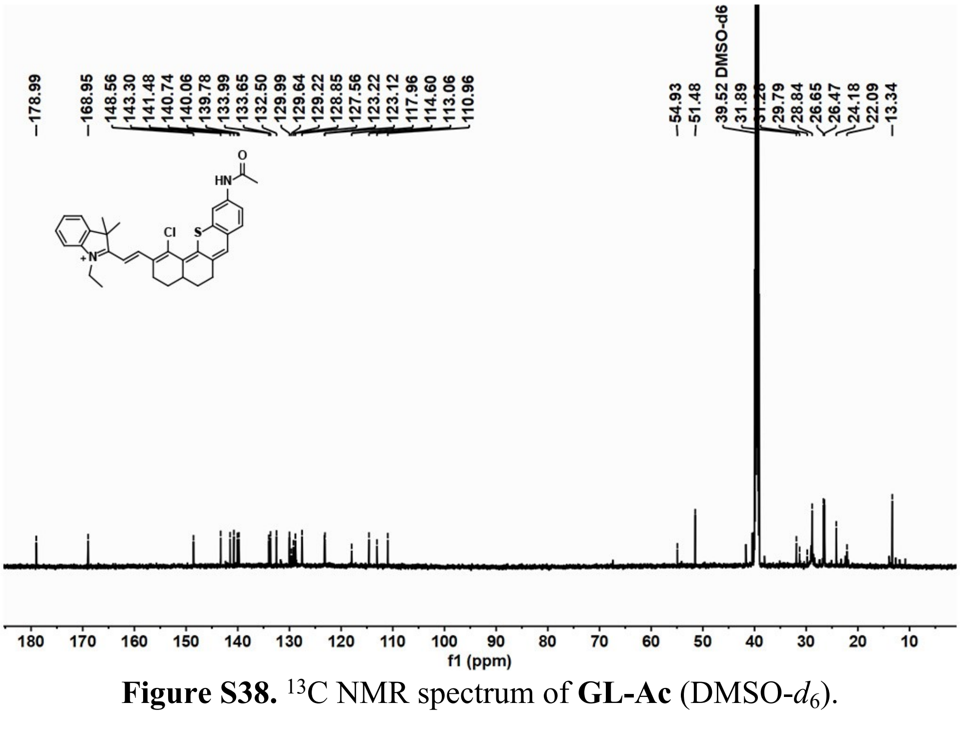

Figure S38. ^{13}C NMR spectrum of **GL-Ac** (DMSO-d_6).

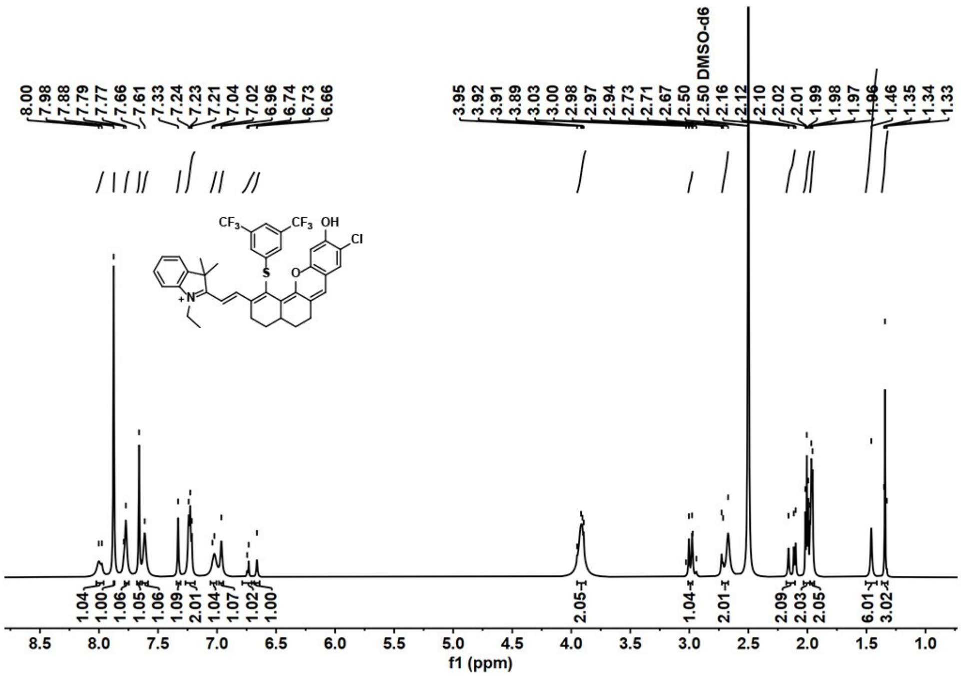

Figure S39. ^{1}H NMR spectrum of **GL-OH** (DMSO-d_6).

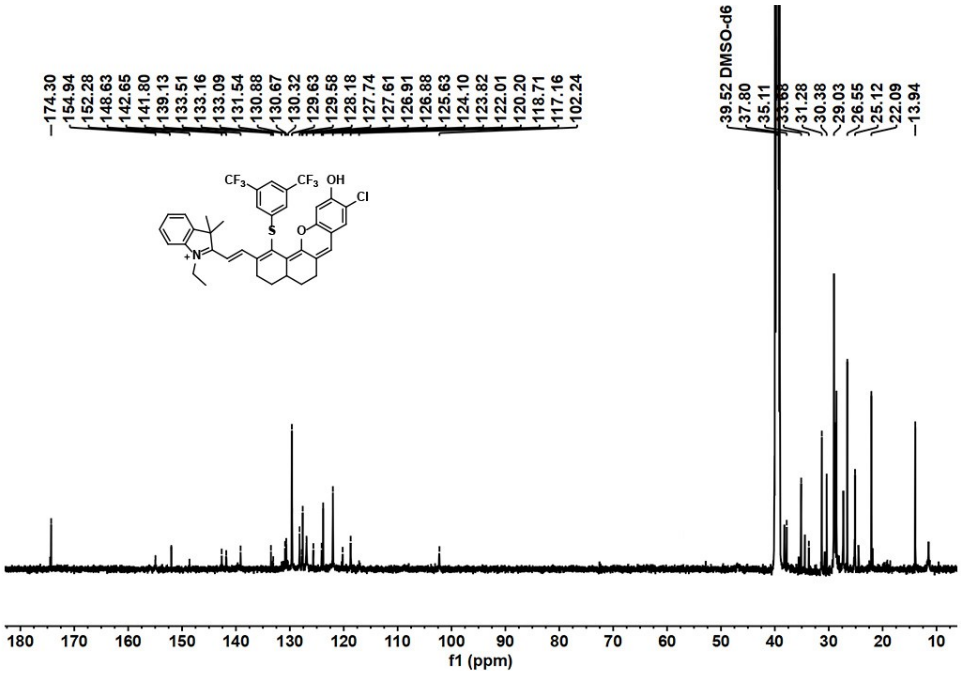

Figure S40. ^{13}C NMR spectrum of **GL-OH** (DMSO-d_6).

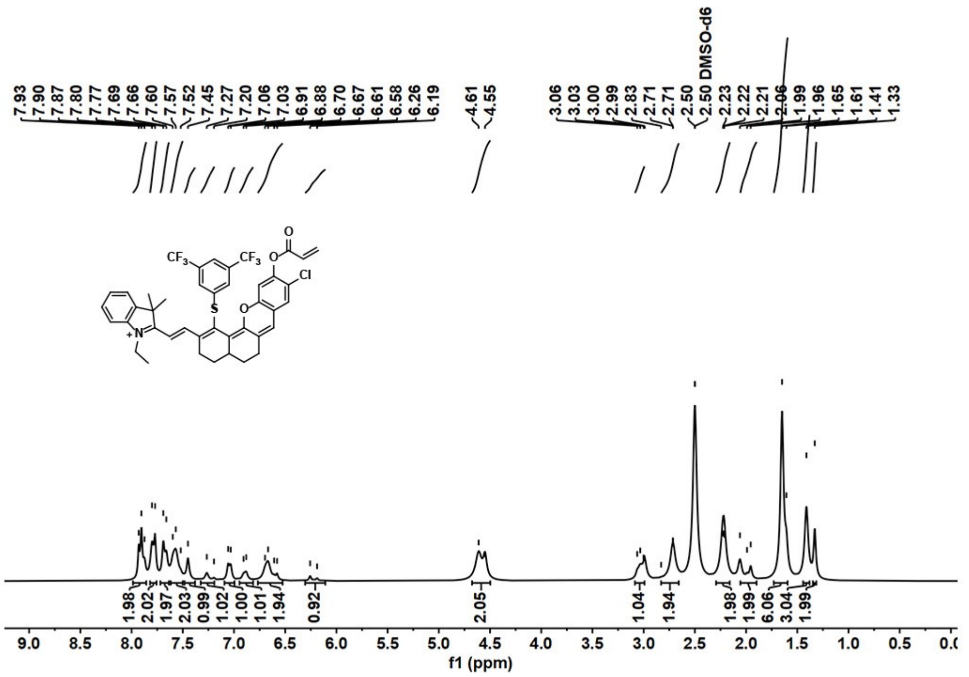

Figure S41. ^{1}H NMR spectrum of **GL-Cys** (DMSO-d_6).

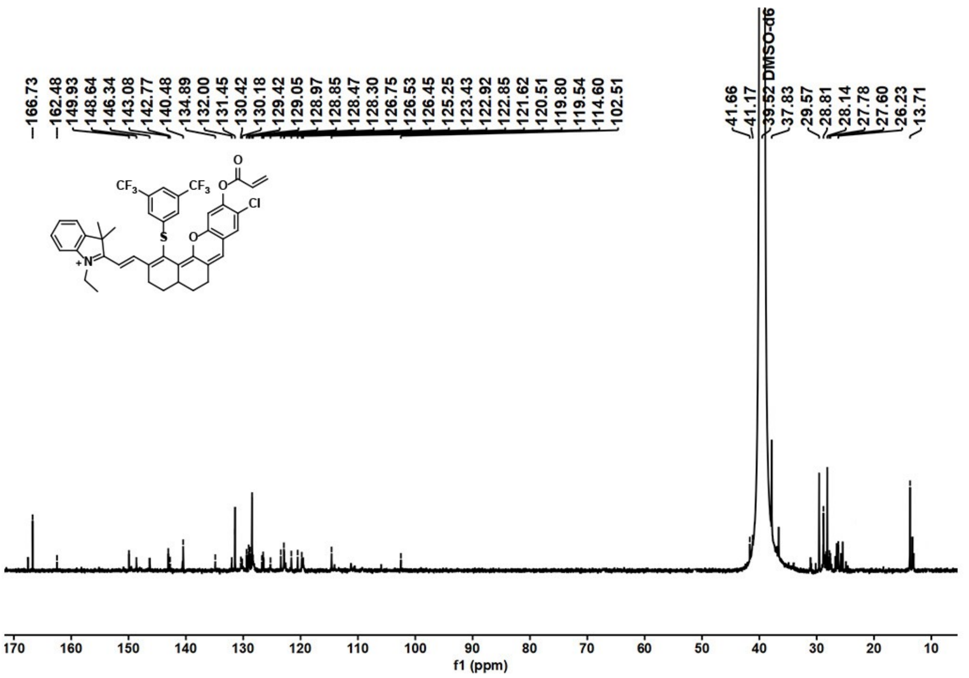

Figure S42. ^{13}C NMR spectrum of **GL-Cys** (DMSO-d_6).
